# Selective autophagy regulates heat stress memory in Arabidopsis by NBR1-mediated targeting of HSP90.1 and ROF1

**DOI:** 10.1080/15548627.2020.1820778

**Published:** 2020-09-24

**Authors:** Venkatesh P. Thirumalaikumar, Michal Gorka, Karina Schulz, Celine Masclaux-Daubresse, Arun Sampathkumar, Aleksandra Skirycz, Richard D. Vierstra, Salma Balazadeh

**Affiliations:** a Max Planck Institute of Molecular Plant Physiology, Potsdam-Golm, Germany; b Institute of Biochemistry and Biology, University of Potsdam, Potsdam-Golm, Germany; c Department of Biology, Washington University in St. Louis, St. Louis, MO, USA; d Institut Jean-Pierre Bourgin, INRA, AgroParisTech, CNRS, Université Paris-Saclay, Versailles, France; e Institute of Biology, Leiden University, Leiden, The Netherlands

**Keywords:** *Arabidopsis thaliana*, heat stress, HSFA2, HSP90.1, NBR1, ROF1, selective autophagy, stress memory, stress recovery

## Abstract

In nature, plants are constantly exposed to many transient, but recurring, stresses. Thus, to complete their life cycles, plants require a dynamic balance between capacities to recover following cessation of stress and maintenance of stress memory. Recently, we uncovered a new functional role for macroautophagy/autophagy in regulating recovery from heat stress (HS) and resetting cellular memory of HS in *Arabidopsis thaliana*. Here, we demonstrated that NBR1 (next to BRCA1 gene 1) plays a crucial role as a receptor for selective autophagy during recovery from HS. Immunoblot analysis and confocal microscopy revealed that levels of the NBR1 protein, NBR1-labeled puncta, and NBR1 activity are all higher during the HS recovery phase than before. Co-immunoprecipitation analysis of proteins interacting with NBR1 and comparative proteomic analysis of an *nbr1*-null mutant and wild-type plants identified 58 proteins as potential novel targets of NBR1. Cellular, biochemical and functional genetic studies confirmed that NBR1 interacts with HSP90.1 (heat shock protein 90.1) and ROF1 (rotamase FKBP 1), a member of the FKBP family, and mediates their degradation by autophagy, which represses the response to HS by attenuating the expression of *HSP* genes regulated by the HSFA2 transcription factor. Accordingly, loss-of-function mutation of *NBR1* resulted in a stronger HS memory phenotype. Together, our results provide new insights into the mechanistic principles by which autophagy regulates plant response to recurrent HS.

**Abbreviations:** AIM: Atg8-interacting motif; ATG: autophagy-related; BiFC: bimolecular fluorescence complementation; ConA: concanamycinA; CoIP: co-immunoprecipitation; DMSO: dimethyl sulfoxide; FKBP: FK506-binding protein; FBPASE: fructose 1,6-bisphosphatase; GFP: green fluorescent protein; HS: heat stress; HSF: heat shock factor; HSFA2: heat shock factor A2; HSP: heat shock protein; HSP90: heat shock protein 90; LC-MS/MS: Liquid chromatography-tandem mass spectrometry; 3-MA: 3-methyladenine; NBR1: next-to-BRCA1; PQC: protein quality control; RFP: red fluorescent protein; ROF1: rotamase FKBP1; TF: transcription factor; TUB: tubulin; UBA: ubiquitin-associated; YFP: yellow fluorescent protein

## Introduction

Temperatures higher than taxa-specific thresholds are damaging, and lethal if sufficiently severe, for all organisms. Among the consequences are the perturbations of cellular metabolism and homeostasis by inducing protein denaturation and aggregation, loss of plasma membrane integrity, and accumulation of reactive oxygen species (ROS) [1–4]. Hence, even mild exposure to high temperatures can cause reductions in biomass production and crop yields, while prolonged exposure (or short exposure to extremely high temperature) leads to cellular proteotoxicity and plant death [2,5]. Like many other environmental stresses, heat stress (HS) is typically transient, but also recurrent and often increases gradually in severity. Therefore, understanding the molecular mechanisms underlying plant responses to repeated HS is essential for any rational improvement of plants in highly dynamic environments.

Exposure to high sub-lethal temperature induces diverse responses that enhance a plant’s ability to maintain cellular homeostasis and survival during the stress [2,3,6–8]. In addition to immediate responses to HS, plants have evolved the ability to remember a previous HS exposure (HS memory), by maintaining some stress-related changes, thereby preparing them for a better response to future HS insults [9–11]. However, responses to stresses are usually accompanied by reductions in growth [12]. Therefore, speedy recovery and reversal of stress-related changes when the stress abates are essential for rapid restoration of “normal” growth (or more accurately maximal growth under the new conditions and constraints imposed by damage caused by the stress) and regained competitiveness. Clearly, to safeguard growth and reproduction in rapidly-changing environments, plants must delicately balance the degree of resetting following stress and maintenance of stress memory. Hence, identifying the components responsible for HS memory and recovery machinery, and elucidating their roles, are essential to improve fundamental understandings of plant physiology and our capacity to enhance a plant's stress resistance.

Recently, accumulating evidence indicates that HS memory and recovery are regulated processes controlled at multiple levels, such as chromatin remodeling, transcriptional activation of heat shock factors (HSFs) and heat shock proteins (HSPs), regulated decay or stabilization of transcripts, and shifts in the turnover of proteins important to protein quality control (PQC) such as HSPs [11,13–18]. One of the key components of HS memory is the heat shock transcription factor HSFA2, which is required for the induction of HS genes and whose sustained elevated levels are crucial for HS memory maintenance [19,20]. Accordingly, *Arabidopsis HSFA2* loss-of-function mutants are dramatically defective in HS memory. At the protein level, HSFA2 interacts with HSP90.1, a major regulator of thermotolerance [21,22]. In mammals, HSP90binds to the co-chaperones immunophilins FKBP5/FKBP51 (FKBP prolyl isomerase 5) and FKBP4/FKBP52 and regulates glucocorticoid receptor activity, which is required for diverse physiological processes, including energy homeostasis.

Interestingly, *Arabidopsis* HSP90.1 interacts with ROF1/AFKBP62 (rotamase FKBP 1), a plant homolog of mammalian FKBP4/FKBP52, and regulates responses to HS [22,23]. Under normal conditions, the HSP90.1-ROF1 complex remains in the cytoplasm, but following exposure to HS, HSFA2 then binds HSP90.1-ROF1, and the resulting complex (HSFA2-HSP90.1-ROF1) translocates to the nucleus. Formation of this complex is putatively required for enhanced transcriptional activity of HSFA2 and continuity of HSP synthesis during HS recovery, thus making the plant more responsive to an imminent recurrence of the HS [22]. Accordingly, *Arabidopsis* overexpressing *ROF1* displays improved HS memory and a sustained increase in expression of HSFA2-regulated genes, whereas HS memory is impaired in *rof1* mutant plants [22,24].

Another pathway that impacts the recovery from HS is autophagy, which is a highly conserved catabolic route among eukaryotes [25]. Autophagy is a multistep process that involves the formation of double-membrane vesicles called autophagosomes that sequester and transport unwanted or damaged cellular material to the lytic compartments (vacuoles in plants) where they are deposited inside as autophagic bodies for efficient degradation and recycling. Formation of autophagosomes upon autophagic induction requires extensive membrane rearrangements and activities provided by a core set of 30 or more autophagy-related (ATG) proteins [26,27]. Recent advances in studies with yeast, mammals, and (to a much lesser extent) plants provide evidence that autophagy can act in a selective manner [28–32], through a wide array of autophagy receptors that specifically recognize damaged or unwanted intracellular constituents (cargos) and target them for autophagic breakdown [30,31,33–36]. These receptors interact with lipidated Atg8-family proteins decorating autophagic membrane through short peptide motifs of two types, called Atg8-interacting motifs (AIMs) and ubiquitin-interacting motifs (UIMs), respectively [36,37].

Autophagy plays an essential role in plant development and responses to environmental cues such as abiotic stresses and interaction with pathogens [25,30,31,38–41]. However, there has been little exploration of the role(s) for selective autophagy in regulating these processes, and only a few autophagy receptors have been functionally characterized in plants. Furthermore, the role of autophagy in response to repeated stresses is poorly understood, as is the involvement of dedicated receptors. 

We recently showed that autophagy lack-of-function mutants are impaired in the degradation of specific HSPs, including HSP90.1, following release from HS, and thus have improved tolerance to future HS [25]. Here, we present mechanistic evidence for the involvement of NBR1, a plant homolog of the mammalian autophagic cargo receptor SQSTM1/p62, in selective autophagy during HS recovery. We demonstrate that NBR1 binds ROF1 and HSP90.1, and mediates their degradation during the HS recovery phase, thereby repressing HSFA2 transcriptional activity, continuity of HSP synthesis, and the enhanced protection to potentially imminent HS. Collectively, our findings unveil the essential role of NBR1 in regulating cellular homeostasis during recovery from HS and illuminate the functional mechanisms through which autophagy regulates responses to repeated HS.

## Results

### NBR1 accumulates during the HS recovery phase and regulates HS memory

**Figure 1.**
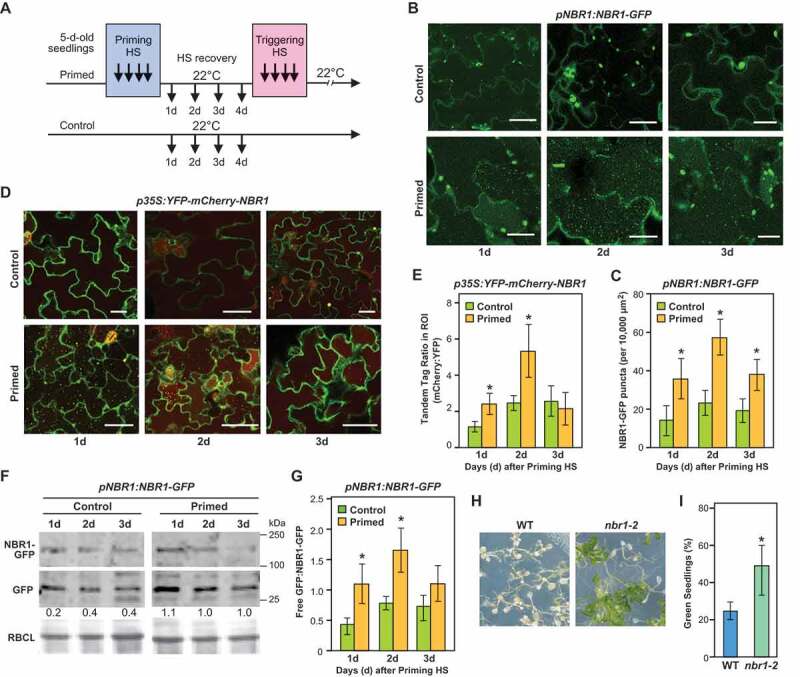
NBR1 accumulates in vacuoles during recovery from heat stress and suppresses HS memory in *Arabidopsis*. (A) Schematic representation of heat stress (HS) regimes applied to probe HS memory. Five-day-old seedlings were subjected to a mild HS of 1.5 h at 37°C, followed by 1.5 h recovery at 22°C and then 45 min at 44°C (hereafter, priming HS). The seedlings were then returned to normal growth conditions for 3 or 4 d, in the HS recovery phase, following which they were again subjected to a severe HS at 44°C for 90 min (triggering HS). Non-heat-treated samples were used as controls. (B) Accumulation of NBR1-GFP puncta during the HS recovery phase. *pNBR1:NBR1-GFP* seedlings were exposed to the priming HS, then normal growth conditions at 22°C. NBR1-GFP fluorescence signals were visualized in cotyledons by fluorescence confocal microscopy 1, 2 and 3 d after cessation of the priming HS, during the recovery phase. Unprimed plants were used as controls. Plants were treated in darkness with 1 μM ConA, an inhibitor of vacuolar H^+^-ATPase activity and proteolytic degradation, 4 h before microscopy observation. Scale bars: 25 µm. (C) Numbers of NBR1-GFP green puncta per frame (10,000 µm^2^ of leaf epidermis section). Data are means ± SD (n = 6). (D) Five-day-old transgenic *Arabidopsis* seedlings expressing *35S:YFP-mCherry-NBR1* were subjected to the priming HS and returned to normal conditions (22°C). NBR1 vacuolar import visualized by higher fluorescence of mCherry is more stable in vacuoles than YFP in primed plants than in untreated control plants during the HS recovery phase after the priming treatment. Scale bars: 50 µm. Data are means ± SD (n = 6). (E) Quantification of NBR1 vacuolar import according to YFP:mCherry signal ratios. (F) Results of immunoblot analysis of autophagic fluxes in primed and unprimed (control) *pNBR1:NBR1-GFP* seedlings at indicated time points in the recovery phase. NBR1-GFP fusion protein and free GFP, detected using anti-GFP antibodies, are indicated. Relative intensities (free GFP:loading control) are shown as numerical values. Ponceau-stained RBCL was used as a loading control (bottom panel). (G) Vertical bar graphs show free-GFP:NBR1-GFP ratios of samples obtained during the HS recovery phase and in control conditions, representative scans of the immunoblots are presented above. Bars represent means (± SD) of three biological replicates. Asterisks in panels C, E, and G indicate significant (*p* ≤ 0.05) differences between samples of plants in control and primed conditions according to Student’s *t*-test. Samples were electrophoresed on the same gel. Full-size images are presented in Figure S7. (H) HS memory phenotypes of *nbr1-2* and wild type (WT) seedlings. Briefly, five-day-old *nbr1-2* and Col-0 WT seedlings were subjected to HS regimes, and their HS memory phenotypes were determined 14 d after triggering HS. Representative images are shown. (I) Percentages of green seedlings (as indicators of seedling survival rates) are shown in bar graphs in the right panel. Data are means ± SD (n = 4). Asterisks indicate significant (*p* ≤ 0.05) differences between WT and *nbr1-2* plants, again according to Student’s *t*-test

Our recent studies revealed an important role of autophagy in recovery from HS [25]. To test whether NBR1 has a role in the selectivity of autophagy during this process, we first examined NBR1 abundance and turnover after release from mild HS and during the recovery phase. To this end, we subjected *Arabidopsis* seedlings to an established HS memory protocol, involving two periods of HS (priming and triggering HS) with an intervening recovery of 4 d (Figure 1A). Firstly, we monitored the abundance of NBR1 during the HS recovery phase using a transgenic line expressing an NBR1-GFP fusion under the control of the *NBR1* promoter (*pNBR1:NBR1-GFP*). Both primed and control (unprimed) plants were exposed to concanamycin A (ConA), an inhibitor of vacuolar acidification that blocks vacuolar proteolysis. As expected, primed plants accumulated a significantly greater number of NBR1-GFP puncta than untreated control plants at all three selected time points during the recovery phase, with a peak at day 2 (Figure 1B,C, S1A-D and Video S1). This increase confirmed that the delivery of NBR1 to the vacuole through autophagy is enhanced during the HS recovery phase. The accumulation of vacuolar NBR1-GFP puncta during the HS recovery phase mimicked that for autophagic bodies [25]. Few NBR1-GFP puncta were detected in the vacuoles of the primed samples in the absence of ConA pretreatment, confirming the active degradation of NBR1 in the vacuole during the HS recovery phase (Figure S1A,B).

We then used a tandem-fluorescence reporter consisting of the acidic pH-sensitive and -stable tags, yellow-fluorescent protein (YFP) and mCherry, respectively, to better assay the involvement of autophagy. In the acidic vacuoles, the YFP fluorescence is quenched, while the mCherry fluorescence should remain largely robust. *35S:YFP-mCherry-NBR1 Arabidopsis* seedlings were subjected to priming HS, then mCherry and YFP fluorescence were visualized by confocal microscopy at selected time points during the HS recovery phase (days 1, 2 and 3) to differentiate cytosolic autophagosomes emitting both mCherry and YFP fluorescence from autophagic bodies only emitting mCherry fluorescence. As shown in Figure 1(D), strong mCherry-mediated fluorescence was observed from the central vacuole of primed samples, but not controls, during the HS recovery phase. The mCherry/YFP ratio was higher in primed plants than in untreated controls, particularly on day 2 during the recovery phase (Figure 1E).

To further examine the autophagy-dependent degradation of NBR1, we conducted GFP-cleavage assays that measure autophagic flux based on the accumulation of free-GFP derived from autophagic substrate-GFP fusion due to relatively high stability of the GFP fragment once inside vacuoles. Quantitative immunoblotting of extracts prepared from primed *pNBR1:NBR1-GFP* seedlings with anti-GFP antibodies revealed a higher accumulation of free-GFP released from NBR1-GFP in the primed samples during the HS recovery phase, as compared to unprimed controls especially in day 1 and 2 of the recovery phase (Figure 1F,G). Finally, we tested HS memory of a *nbr1-2* null mutant [30]. *nbr1-2* plants showed enhanced HS memory compared to wild-type (Col-0) plants, indicating a functional role for NBR1 in HS memory (Figure 1H, I). Under control conditions, the growth of the mutants did not differ from wild type (WT) but did show a reduced basal thermotolerance, as determined by exposing plants to a single severe HS (44°C for 30 min) and measuring their subsequent survival (Figure S1E-G), in agreement with previous findings [42]. Our results highlight a distinct role for NBR1 in response to single and repeated HS. Together, the data showed that NBR1 hyper-accumulates and is dynamically turned over during the HS recovery phase and that it negatively regulates HS memory.

### NBR1 is active as an autophagic receptor during HS recovery

**Figure 2.**
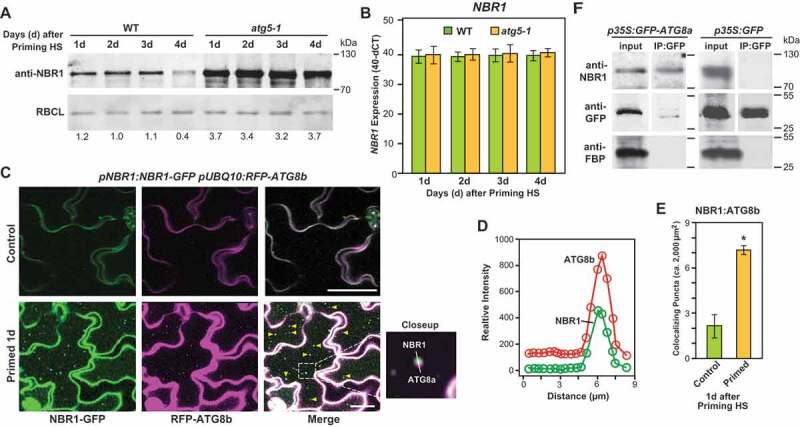
NBR1 associates with autophagy during the HS memory. (A) Results of immunodetection of NBR1 during the HS recovery phase in *atg5-1* and Col-0 wild-type (WT) seedlings using anti-NBR1 antibodies (Agrisera AS142805). RBCL served as the loading control (bottom panels). Relative intensities (NBR1:loading control) are shown as numerical values. Samples were electrophoresed on the same gel. (B) Expression levels (determined by qRT‐PCR) of *NBR1* in WT and *atg5-1* mutant seedlings at indicated time points (1, 2, 3 and 4 d) in the HS recovery phase. Values are differences between an arbitrary value of 40 and dCt, calculated as described [11]. Data are means ± SD (*n* = 4, where *n* represents independently performed experiments). (C) Colocalization of NBR1-GFP with RFP-ATG8b autophagosomes using *pNBR1:NBR1-GFP* and *pUBQ10:RFP-ATG8b* transgenic lines. Microscopy images were taken after the priming treatment during the HS recovery phase (1 d). Scale bars: 25 µm. (D) Double intensity plots of colocalizing NBR1-GFP puncta and RFP-ATG8b-labeled autophagosomes. The green and red lines represent relative intensities of NBR1-GFP and RFP-ATG8b signals, respectively. (E) Numbers of colocalizing NBR1-GFP puncta and RFP-labeled autophagosomes, per 2,000 µm^2^ of leaf epidermis section. Data are means ± SD (n = 3). Asterisks indicate significant (*p* ≤ 0.05) differences between samples of plants subjected to control and primed conditions according to Student’s *t*-test. (F) Qualitative results of the CoIP experiment *GFP-ATG8a* seedlings were subjected to priming heat stress treatment and samples were harvested 2 d into the HS recovery phase. Total proteins were extracted and immunoprecipitated with anti-GFP antibody beads. Immunoprecipitates (CoIP) obtained with anti-GFP beads and total protein extracts were immunoblotted with appropriate antisera, as indicated in the figure. Full-size images are presented in Figure S7

Although various studies revealed that NBR1 is a major cargo receptor in autophagy, the mechanistic details of NBR1-mediated selective autophagy are still largely unknown [30,41–43]. To assess whether NBR1 functions as an autophagy receptor during HS recovery, we first examined the level of NBR1 protein in autophagy loss-of-function (*atg5-1* and *atg18a-2*) mutants [11,44]. Immunoblot analysis showed that NBR1 accumulates to substantially higher levels in these mutants than in WT plants during the HS recovery phase (Figure 2A and S2). As the levels of the *NBR1* transcript did not differ between *atg5-1* mutant and WT, it appeared that the increased accumulation of NBR1 protein is not transcriptionally driven and likely caused the participation of NBR1 as an autophagic cargo receptor during HS recovery (Figure 2B).

NBR1 contains an AIM that helps tether substrates into the autophagosomes through its interaction with lipidated ATG8 embedded in the engulfing membranes. To assess the association of NBR1 with ATG8-decorated autophagic vesicles during recovery from HS, we monitored co-localization of NBR1 and ATG8B using transgenic lines co-expressing *NBR1-GFP* and *RFP-ATG8b* generated by co-transformation of the *pNBR1:NBR1-GFP* and *pUBQ10:RFP-ATG8b* transgenes. As shown in Figure 2(C,D) (and Video S2), GFP and RFP fluorescence co-localized in punctate structures within cytoplasm and vacuole that likely reflected association of both NBR1 and ATG8B with the autophagosomes and autophagic bodies, respectively. The co-localization ratio was significantly higher in the primed tissues than in untreated controls (Figure 2E). As predicted for autophagic bodies, the abundance of vacuolar puncta decorated with RFP-ATG8b and GFP-NBR1 was higher upon ConA treatment (Video S2).

We also tested the interaction between ATG8a and NBR1 during HS recovery by pulldown assays. Protein extracts from *35S:GFP-ATG8a* and *35S:GFP* seedlings were immunoprecipitated with anti-GFP antibodies and immunoblotted with anti-NBR1 antibodies. As shown in (Figure 2F), NBR1 immunoprecipitated along with GFP-ATG8a (left panel) but not GFP alone (right panel), indicating a direct interaction between NBR1 and ATG8a. To further verify the specificity of the interaction, we used antibodies that recognizes a cytosolic housekeeping protein, FBP/FBPASE (fructose 1,6-bisphosphatase); no co-immunoprecipitation of FBP with GFP-ATG8a was observed (Figure 2F. Collectively, our results suggest that NBR1 acts as a selective autophagy receptor during the HS recovery.

### Identification of NBR1 cargoes during the HS recovery phase

**Figure 3.**
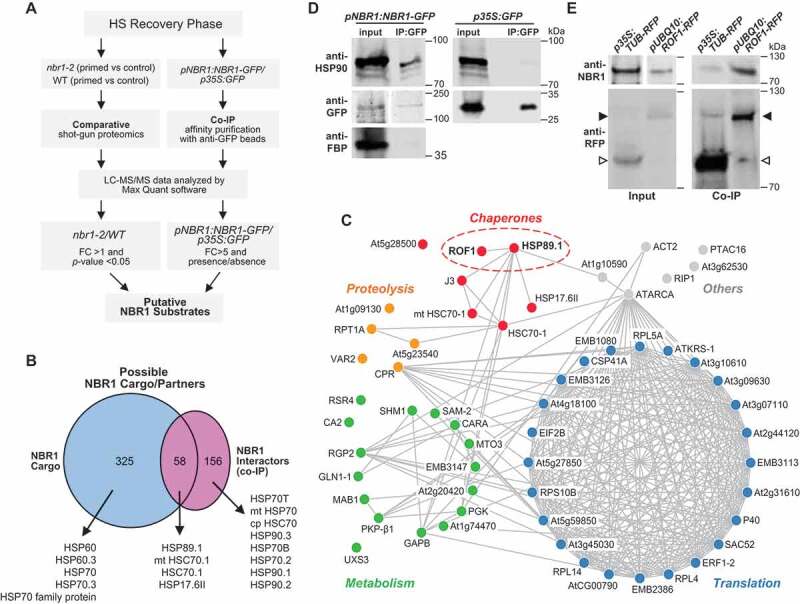
Identification of NBR1 substrate proteins during the HS recovery phase. (A) Schematic representation of the proteomic workflow with two complementary approaches to identify NBR1 substrate proteins during the HS recovery phase. (B) Venn diagram summarizing the proteins overlapping between NBR1 cargos and NBR1 interactor datasets during the HS recovery phase. Notably, there is an overlapping set of 58 proteins present in both datasets. All heat shock proteins that were associated with NBR1 are indicated by arrowheads. (C) Results of functional network analysis, visualized using Cytoscape (https://cytoscape.org/). Nodes represent putative NBR1 substrate proteins. Blue, red, green, and yellow represent translation, chaperones, metabolic enzymes, and proteolysis, respectively. (D) HSP90 co-immunoprecipitates with NBR1. *pNBR1:NBR1-GFP* seedlings were subjected to priming HS treatment and samples were harvested 2 d into the HS recovery phase. Total proteins were extracted and immunoprecipitated with anti-GFP antibody beads. Immunoprecipitates (IP) and total protein extracts (input) were immunoblotted with appropriate antibodies as described in the Figure. (E) ROF1 co-immunoprecipitates with NBR1. *pUBQ10:ROF1-RFP* and *35S:TUB-RFP* (a negative control) seedlings were subjected to priming HS treatment and samples were harvested 2 d into the HS recovery phase. Total proteins were extracted and immuno-precipitated with anti-RFP antibody beads (Chromotek). Immunoprecipitates (IP) and total protein extracts (input) were immunoblotted with appropriate antisera as described in the Figure. Full-size images are presented in Figure S7

To further define the roles of NBR1 in selective autophagy and cargo recruitment during HS recovery, we attempted to identify NBR1-binding partners using two complementary approaches (Figure 3A and Datasets S1-S4 and Table S1). In one, we performed co-immunoprecipitation (CoIP) assays with anti-GFP antibody-tagged beads using total extracts prepared from *pNBR1:NBR1-GFP* and *35S:GFP* (negative control) seedlings harvested at day 1 during the HS recovery phase, followed by liquid chromatograph-mass spectrometry (LC-MS/MS) detection of the associated proteins. After normalization and excluding GFP-interacting proteins, 213 proteins were identified as putative NBR1 partners (Datasets S2, S4A and S4B). In the second approach, we compared the total detectable proteomes of *nbr1-2* and WT seedlings by LC-MS/MS during the HS recovery phase (days 1, 2 and 3). In total, we identified 1,898 proteins, of which 64, 165 and 198 were significantly more abundant following priming in the *nbr1-2 *mutants than in WT seedlings (*p* ≤ 0.05) at days 1, 2 and 3, respectively, and thus were designated as possible NBR1 interactors (Datasets S1 and S3).

Next, to identify NBR1 substrates during the HS recovery phase, we compared the list of possible NBR1 cargo candidates with the list of putative NBR1 interactors, resulting in a collection of 58 common proteins, which we designated as high-confidence NBR1 interactors (Figure 3B). Functional network analysis using the STRING database (https://string-db.org/) revealed that the 58 proteins were part of several interaction networks related to translation, metabolism, proteolysis, and chaperones. Included were multiple stress-responsive proteins, such as UXS3 (UDP-GLUCURONIC ACID DECARBOXYLASE 3), SAM-2 (S-ADENOSYLMETHIONINE SYNTHETASE 2), EMB3147 (EMBRYO DEFECTIVE 3147), and GLN1-1 (ARABIDOPSIS GLUTAMINE SYNTHASE 1) (yellow nodes in Figure 3C), metabolic enzymes, such as RPT1A (REGULATORY PARTICLE TRIPLE-A 1A), CA2 (BETA CARBONIC ANHYDRASE 2), and RSR4 (*ARABIDOPSIS THALIANA* PYRIDOXINE BIOSYNTHESIS 1.3) (blue nodes in Figure 3C) and proteins involved in translation/ribosome subunits, such as RPL5A, RPL10A/SAC52 (60S RIBOSOMAL PROTEINs), and RP40 and RPS10B (40S RIBOSOMAL PROTEINs) (red nodes in Figure 3C). Notably, several in the list were molecular chaperones and co-chaperones, such as HEAT SHOCK PROTEIN 90.3 (HSP89.1), ROTAMASE 1 (ROF1), and HEAT SHOCK PROTEIN 17.6 II (HSP17.6II) (green nodes in Figure 3C).

Among the list were HSP90 chaperones and ROF1, which were selected for further functional analysis due to their previously reported significance in regulating HS memory [22,45]. To follow up, we confirmed the interactions between NBR1 and HSP90.1 and ROF1 during HS recovery (2 d) by CoIP/immunoblot analysis. As demonstrated in (Figure 3D), we successfully recovered members of the HSP90 family in extracts from *pNBR1:NBR1-GFP* seedlings but not from *35S:GFP* seedlings using anti-GFP antibodies for the pulldowns and followed by immunoblotting with anti-HSP90 antibodies for detection. To further verify the specificity, we also tested FBP, which was not recovered in the immunoprecipitates with anti-GFP antibodies (Figure 3D). To confirm the interaction between NBR1 and ROF1 during the HS recovery, protein extracts prepared from *pUBQ10:ROF1-RFP* and *35S:TUB-RFP* seedlings (as a negative control) after 2 d of recovery were immunoprecipitated with an anti-RFP monoclonal antibody and immunoblotted with anti-NBR1 antibodies. As demonstrated in Figure 3E, NBR1 co-immunoprecipitated with ROF1-RFP but not with RFP alone, indicating a direct interaction between NBR1 and ROF1.

### NBR1 associates with ROF1 and HSP90.1 and regulates their degradation during HS recovery

To confirm the role of NBR1 as a receptor for the selective autophagic degradation of ROF1 and HSP90 during the HS recovery phase, we performed a series of cellular, biochemical and molecular analyses. While the *Arabidopsis* genome encodes four cytosolic HSP90 isoforms, we focused on HSP90.1, given its reported connection to HS memory [22].

**Figure 4.**
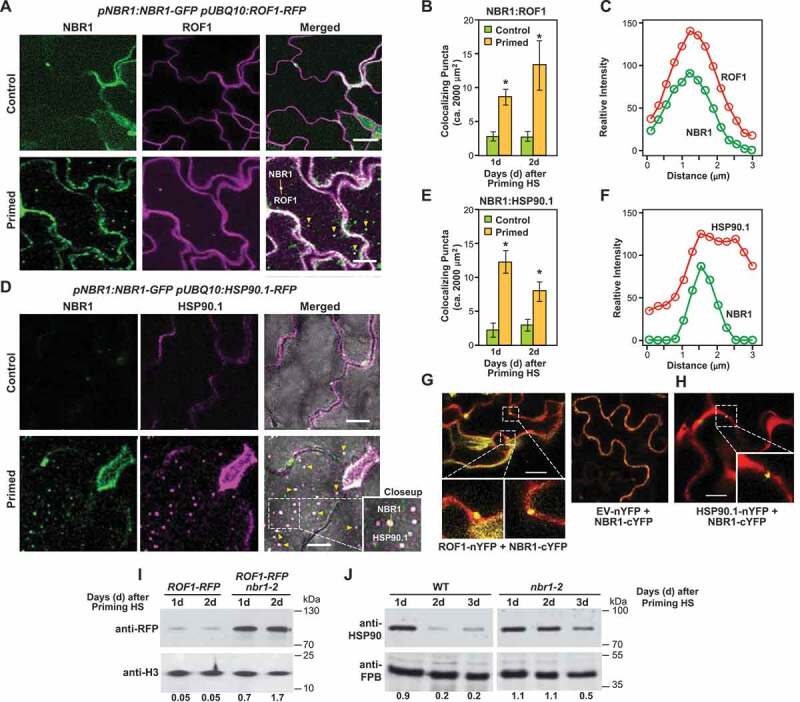
NBR1 targets ROF1 and HSP90 during recovery from HS. (A) NBR1-GFP and ROF1-RFP colocalized in *pNBR1:NBR1-GFP*/*pUBQ10:ROF1-RFP* transgenic lines after heat stress priming HS (2 d). Representative microscopy images 2 d after priming. Scale bars: 25 µm. (B) Numbers of colocalizing NBR1 and ROF1 puncta per frame (2,000 µm^2^ of leaf epidermis section), assessed by counting the white puncta (green + magenta). Data are means ± SD (n = 3). Asterisks indicate significant (*p* ≤ 0.05) differences between samples of plants in control and primed conditions according to Student’s *t*-test. Scale bars: 10 µm. (C) Intensity plots for colocalizing NBR1-GFP (green) and ROF1-RFP (red) puncta 2 d after thermopriming. (D) NBR1-GFP and HSP90.1-RFP co-localized in *pNBR1:NBR1-GFP*/*pUBQ10:HSP90.1-RFP* transgenic lines. Microscopy images were captured after the priming treatment during the HS recovery phase (2 d). Representative microscopy images after priming are shown. Scale bars: 25 µm. (E) Numbers of colocalizing NBR1 and HSP90.1 puncta, assessed by counting white puncta (green + magenta) per frame (2,000 µm^2^ of leaf epidermis section). Data are means ± SD (n = 3). Asterisks indicate significant (*p* ≤ 0.05) differences between samples of plants in control and primed conditions according to Student’s *t*-test. (F) Intensity plots for colocalizing NBR1-GFP (green) and HSP90.1-RFP (red) puncta 2 d after HS priming. (G) Results of BiFC with agro-infiltrated *Nicotiana benthamiana* leaves showing interaction in the epidermal layer between NBR1 and ROF1. cYFP and nYFP refer to C-terminal YFP fragment and N-terminal YFP fragment, respectively. Red indicates a cytosolic marker, and white boxes indicate the interaction signal. GUS-YFP was used as a negative control. Scale bars: 50 µm. (H) Results of BiFC with agro-infiltrated *N. benthamiana* leaves showing interaction between NBR1 and HSP90.1 in the epidermal layer. GUS-nYFP was used as a negative control. Scale bars: 20 µm. (I) Results of immunodetection of ROF1-RFP during the HS recovery phase in *pUBQ10:ROF1-RFP* and *pUBQ10:ROF1-RFP*/*nbr1-2* seedlings using an anti-RFP antibody (Chromotek, 6G6; 1:1,000). Antibodies against histone H3 (αH3, detected using Abcam, ab1791; 1:5,000) was used to confirm near equal protein loading. Relative intensities (ROF1-RFP/loading control, and HSP90/loading control) are shown as numerical values. Samples were electrophoresed on the same gel. Full-size images are presented in Figure S7. (J) Results of immunodetection of HSP90.1 during the HS recovery phase in wild type (left panel) and *nbr1-2* (right panel) seedlings using anti-HSP90.1 antibody (Agrisera, AS08346; 1:3,000). FBP was detected as a loading control with antibodies provided by Agrisera (AS04043; 1:5,000). Relative intensities (HSP90.1/loading control) are shown as numerical values. Samples were electrophoresed on the same gel. Full-size images are presented in Figure S7

First, co-localization of NBR1 with ROF1 and HSP90 was visualized and quantified using *pNBR1:NBR1-GFP*/*pUBQ10:ROF1-RFP* and *pNBR1:NBR1-GFP*/*pUBQ10:HSP90.1-RFP* transgenic lines generated by introgression. Both ROF1 (RFP) and HSP90.1 (RFP) co-localized with NBR1 (GFP) (Figure 4A-F, S3A and Video S3 and S4). Especially notable was the detection of NBR1 with either ROF1 or HSP90.1 in vacuolar puncta that likely represented autophagic bodies. Moreover, the co-localization was significantly stronger in primed cells at both selected time points (1 and 2 d into the HS recovery phase) than in control (unprimed) cells (Figure 4B-E). To verify the interaction between NBR1 and either ROF1 or HSP90.1, we employed bimolecular fluorescence complementation (BiFC) assays in which each protein was fused to the N-terminal or C-terminal halves of YFP. As shown in (Figure 4G,H, successful restoration of the YFP signal was generated in *Nicotiana benthamiana* leaves when NBR1-cYFP was co-expressed with either ROF1-nYFP or HSP90.1-nYFP.

If NBR1 acts as a receptor for autophagic degradation of ROF1 and HSP90, levels of ROF1 and HSP90 proteins should be higher in *nbr1-2* null mutant than in WT (if there are no counteracting processes). For HSP90.1, we compared its levels in WT and *nbr1-2* seedlings using general anti-HSP90 antibodies. For ROF1 levels, we compared its levels in WT and *nbr1-2* seedlings, also expressing the ROF1-RFP fusion using the anti-RFP antibody for immunodetection (Figure S3B). Higher levels of both HSP90 and ROF1 were detected in the *nbr1-2* seedlings compared to WT at days 1 and 2 during the HS recovery phase. By contrast, levels of the nuclear and cytosolic housekeeping proteins histone H3 and FBP, respectively, did not differ between WT and mutant at any of the selected time points, indicating specificity of ROF1 and HSP90 accumulation in *nbr1-2* plants (Figure 4I,J). Moreover, *ROF1* and *HSP90.1* transcript levels did not differ between *nbr1-2* mutant and WT plants, implying that their higher protein accumulation was independent of its transcription (Figure S3D,E).

**Figure 5.**
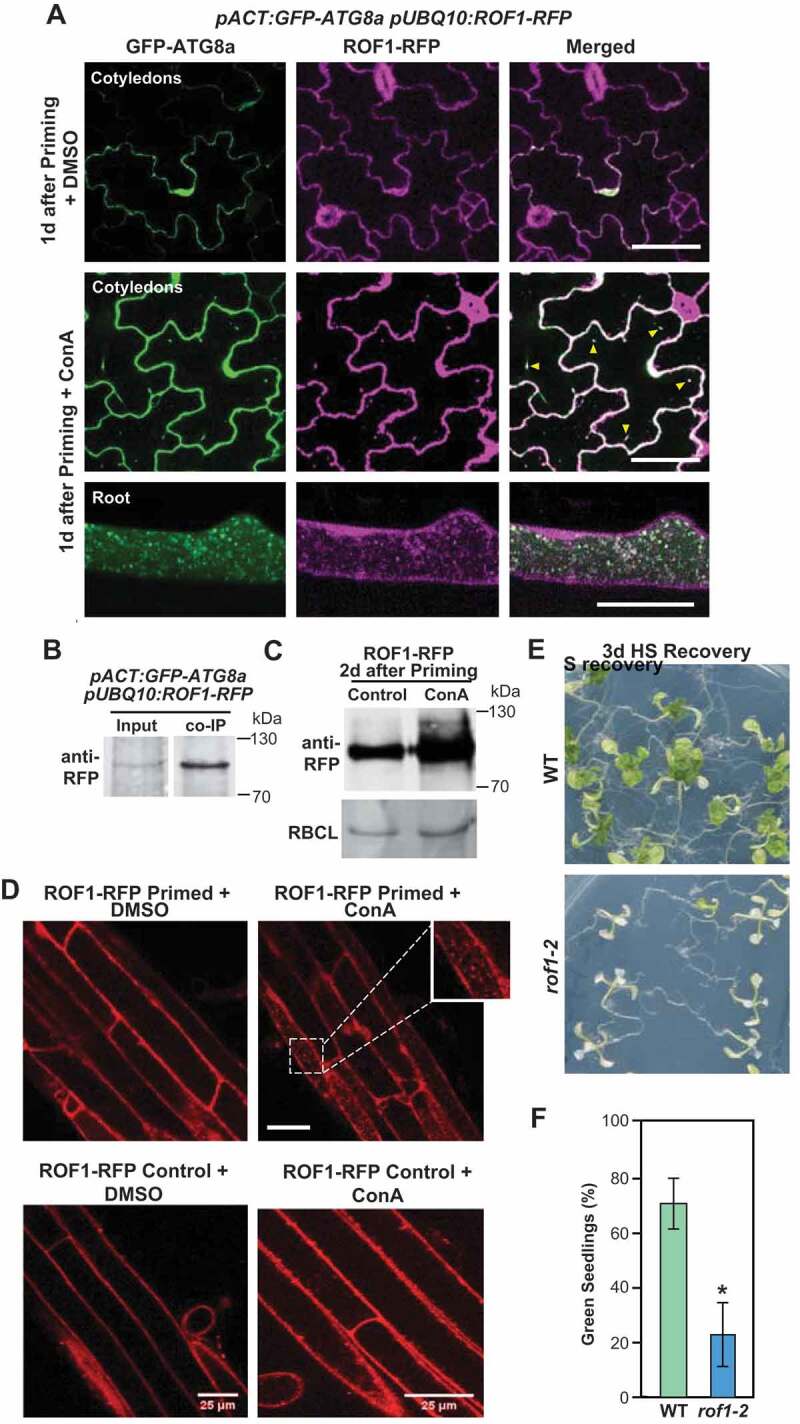
Autophagy is involved in the degradation of ROF1 during HS memory. (A) GFP-ATG8a and ROF1-RFP colocalized after HS priming (1 d) in *pACT:GFP-ATG8a*/*pUBQ10:ROF1-RFP* transgenic plants (cotyledons, upper panel; roots, lower panel). Representative microscopy images are shown. Scale bars: 50 µm. (B) ROF1 co-immunoprecipitates with ATG8. *pACT:GFP-ATG8a*/*pUBQ10:ROF1-RFP* seedlings were subjected to priming HS and samples were harvested 2 d into the HS recovery phase. Total proteins were extracted and immunoprecipitated with anti-GFP antibody beads. Immunoprecipitates (IP) and total protein extracts were immunoblotted with an anti-RFP antibody (Chromotek, 6G6). (C) Higher accumulation of ROF1-RFP during the HS recovery phase (2 d after priming) upon treatment with ConA compared with a DMSO control. ROF1-RFP was detected in the seedlings of *pUBQ10:ROF1-RFP*/Col-0 by immunoblotting using an anti-RFP antibody (Chromotek, 6G6; 1:1,000). RBCL detected by Ponceau-staining was used as the loading control (bottom panel). Relative intensities (RFP:loading control) are shown as numerical values. Full-size images are presented in Figure S7. (D) Presence of ROF1-RFP in the central vacuole during the HS recovery phase. Accumulation of ROF1-RFP in *pUBQ10:ROF1-RFP* seedlings 2 d after priming HS treatment or control (unprimed) was assessed in roots (differentiation zone) by fluorescence confocal microscopy following DMSO control and ConA treatment. Scale bars: 25 µm. (E) HS memory phenotypes of *rof1-2* and Col-0 wild type (WT) seedlings. Briefly, 5-d-old *rof1-2* and Col-0 WT seedlings were subjected to HS regimes to explore HS memory as shown in Figure 1A. Phenotypes were determined 12 d after triggering HS. Representative images are shown. (F) Percentages of green seedlings (indicating seedling survival rates) are shown in bar graphs in the right panel. Data are means ± SD (n = 4 sets of 25 seedlings). Asterisks indicate significant (*p* ≤ 0.05) differences between Col-0 and *rof1-2* plants according to Student’s *t*-test

Confocal time-lapse imaging revealed dynamic movement of ROF1-RFP and HSP90.1-RFP in the vacuole (Figure S3F,G). In particular, higher levels of ROF1-RFP and HSP90.1-RFP were detected in vacuoles of WT following treatment with ConA (Figure 5B, S3H and Video S6, S7), whereas their levels were lower in the *nbr1-2* mutant (Figure S3I,J), confirming that NBR1 is required for the delivery of ROF1 and HSP90.1 to the vacuole during the recovery phase. We also tested the flux of free-RFP (as an indicator of autophagy-dependent protein degradation) by immunoblot analysis of *pUBQ10:ROF1-RFP*/*nbr1-2* and *pUBQ10:ROF1-RFP*/*Col-0* seedlings. As shown in Figure S3K, free-RFP levels were lower in *ROF1-RFP*/*nbr1-2* plants than in *ROF1-RFP* plants.

NBR1 binds ubiquitinated proteins *via* its C-terminal ubiquitin-binding domains (UBA) [41,46]. To test whether the ubiquitination of ROF1 or HSP90.1 is a prerequisite for their interaction with NBR1, we employed co-localization and BiFC assays in *Nicotiana benthamiana* leaves using an NBR1-∆UBA (deletion of both UBA domains) variant. As shown in Figure S4A-D, NBR1-∆UBA still co-localized and interacted with both HSP90.1 and ROF1, indicating that NBR1 binds these substrates independent of ubiquitination. Consistent with these observations, we found that a substantial portion of ROF1 and HSP90.1 accumulating in *nbr1-2* seedlings remained in the soluble and not aggregated protein fractions (Figure S4E,F). Collectively, these data provide convincing evidence that non-aggregated ROF1 and HSP90.1 are selectively degraded, without ubiquitination, by NBR1 for autophagic degradation during HS recovery.

### Autophagic degradation of ROF1 negatively impacts HS memory

We previously reported that autophagy promotes the turnover of HSP90 during HS recovery [25]. To test whether ROF1 is also degraded by autophagy, we generated a *pACT:GFP-ATG8a pUBQ10:ROF1-RFP* transgenic line and checked the co-localization and co-immunoprecipitation of ROF1-RFP with GFP-ATG8a during HS recovery. Confocal microscopy analyses revealed that ATG8a (green puncta) and ROF1 (red puncta) co-localized in the vacuole, which became more obvious upon ConA treatment (Figure 5A and Video S5A and S5B). Moreover, CoIP using anti-GFP antibody beads demonstrated that GFP-ATG8a interacts with ROF1-RFP *in planta* (Figure 5B). Furthermore, treatment with ConA resulted in significantly increased vacuolar levels of ROF1 (Figure 5C,D, and Video S6), indicating that autophagy is involved in ROF1 degradation.

Additionally, we generated a line expressing *pUBQ10:ROF1-mRFP* in the *nbr1-2, atg5-1* double null background and assessed the delivery of ROF1 into the vacuole during the HS recovery. Confocal microscopy detected ROF1 in the vacuoles of WT, but not in those from the *nbr1-2/atg5-1* mutant (Figure S5A). Together, our data support the notion that NBR1-mediated autophagy targets ROF1 and HSP90.1 during HS recovery. Degradation of ROF1 and HSP90.1 by autophagy during recovery from HS appears to be a plausible mechanism for resetting the physiology of *Arabidopsis* post-stress and restoration of previous cellular energy states, at the expense of a weaker response to future stress. Accordingly, mutants lacking ROF1 (*rof1-2*) (Figure S5B,C) had weaker HS memory than WT (Figure 5E).

### NBR1 deficiency enhances expression of HSFA2 target genes during HS recovery

**Figure 6.**
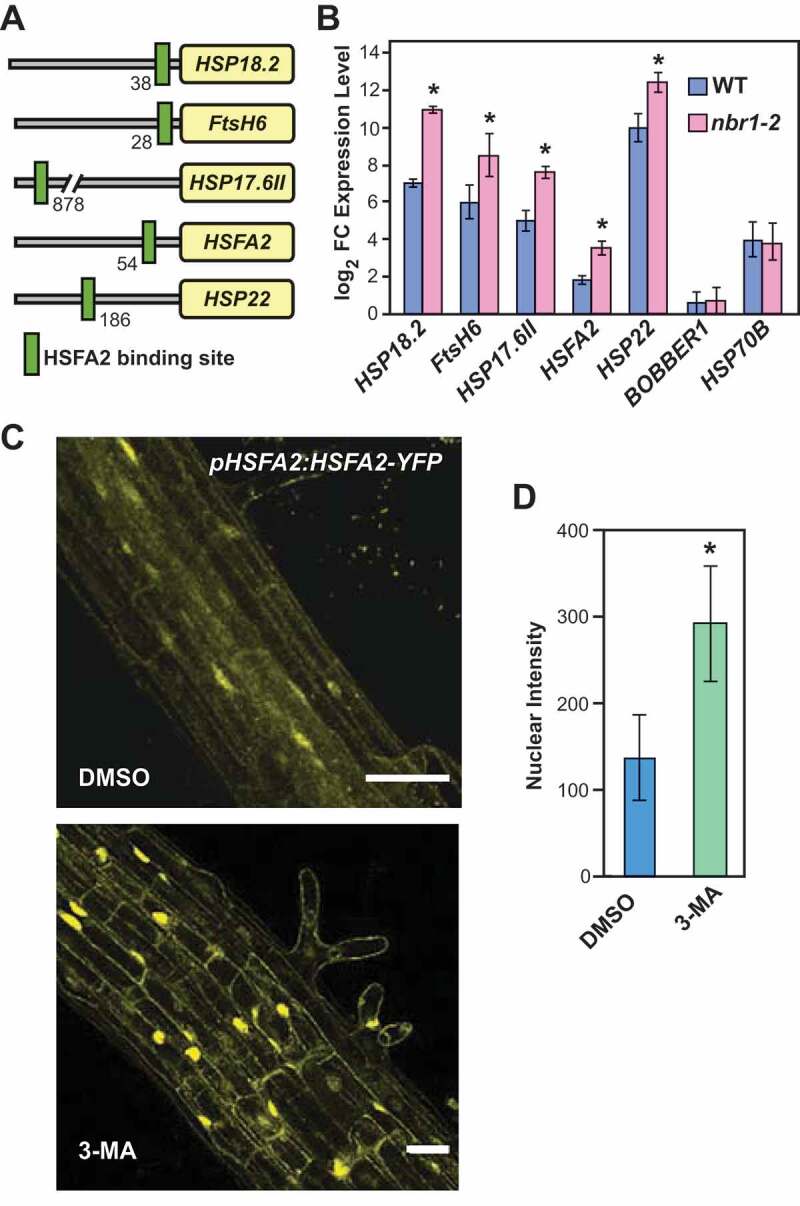
NBR1 deficiency results in enhanced HSFA2 transcriptional activity. (A) Schematic presentation of the heat shock element (HSE) position in promoters of HSFA2 target genes. (B) Results of qRT-PCR expression analysis of *HSFA2* and its target genes in WT and the *nbr1-2* mutant. The Y-axis indicates the expression ratio (log2 fold-changes) of genes in primed (2 d into the recovery phase) compared to untreated controls. Note: the expression of HSFA2 target genes is higher in the *nbr1-2* mutants than in WT. (C) Inhibition of autophagosome formation (by 3-MA treatment) induces nuclear accumulation of HSFA2. Roots (differentiation zone) of *pHSFA2:HSFA2-YFP* seedlings either treated with 5 mM 3-MA dissolved in DMSO or DMSO alone (as control) were visualized for the nuclear localization of HSFA2-YFP by fluorescence confocal microscopy at 2 d into HS recovery Scale bars: 50 µm. (D) The quantification of HSFA2-YFP nuclear intensities was analyzed using IMARIS (https://imaris.oxinst.com/Bitplane)

Sustained high expression of several HS-related proteins and HSPs during the HS recovery phase requires HSFA2 [20,47,48]. In *Arabidopsis*, nuclear localization of HSP90.1 and ROF1 after HS reportedly coincides with increases in transcriptional activation of genes regulated by HSFA2. Therefore, we tested if the higher levels of ROF1 and HSP90 in the *nbr1-2* mutant lead to increased expression of *HSFA2* and its target loci. Based on the literature [20,47] and in-house experiments, we compiled a list of HSFA2 impacted genes and examined their promoter regions for the presence of the HSFA2 core binding site [47]; all contained the core HSFA2-binding element (Figure 6A). Transcript analysis by qRT-PCR revealed significantly higher expression of HSFA2 target genes in *nbr1-2* than in WT plants during recovery from HS (2 d) (Figure 6(B)). To demonstrate the specificity of the response, we tested the expression of two other heat-induced genes not regulated by HSFA2 (*BOBBER1* and *HSP70B* [49,50]) in *nbr1-2* mutant and WT plants. As shown in Figure 6(B), transcript abundances of both genes remained indistinguishable between *nbr1-2* and WT during the recovery from HS, indicating that the induction of HSFA2 target genes in the *nbr1-2* mutant is not part of a general activation of heat response pathways.

**Figure 7.**
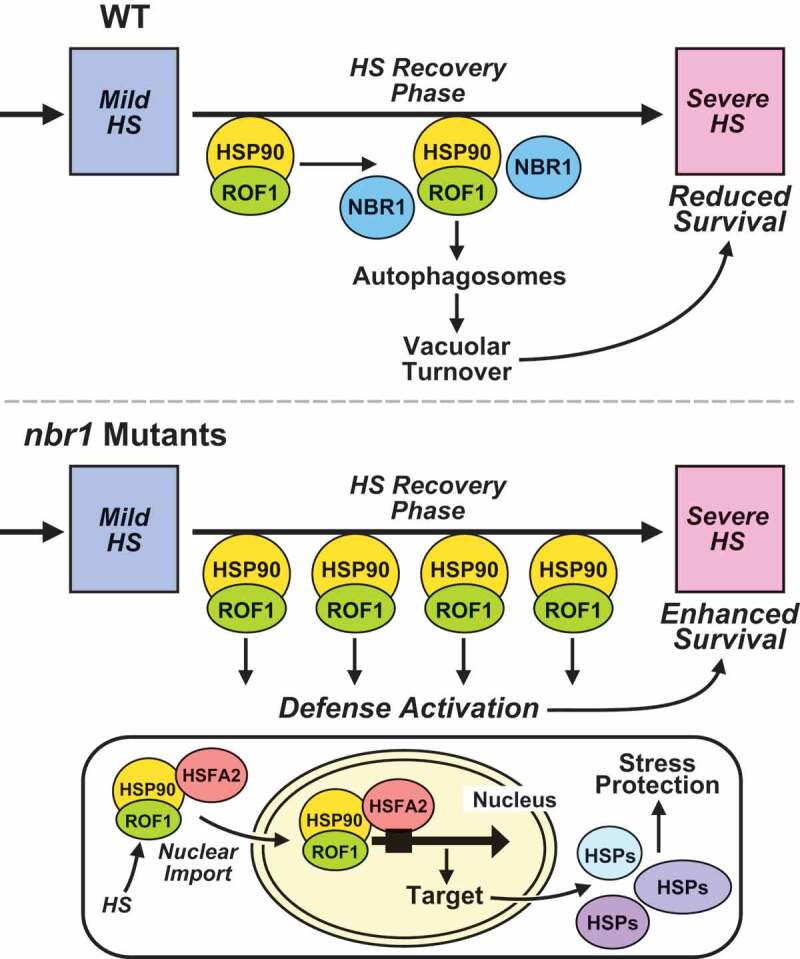
Model for NBR1-mediated regulation of HS memory in *Arabidopsis thaliana*. Proposed model for the role of NBR1-mediated selective autophagy in recovery from HS. Upper panel: Priming HS induces increases in abundance of ROF1 and HSP90.1 proteins (essential components of the HS response machinery). During recovery from HS, NBR1 markedly accumulates, interacts with ROF1 and HSP90.1, and mediates their selective degradation by autophagy. Hence, HS-responsive processes controlled by HSP90.1-ROF1 are impaired, including expression of HSFA2-target genes during the HS recovery phase and subsequently responses to the next severe HS (manifested by lower survival rates). Lower panel: Situation in *nbr1-KO* (*nbr1-2*) mutants: Lack of NBR1 results in sustained high levels of ROF1 and HSP90.1 during the HS recovery phase, and thus enhanced HSFA2 activity (manifested by higher expression of the HSFA2 target genes) during HS recovery. Taken together, lack of NBR1 results in enhanced HS memory capacity and better protection from severe post-memory heat stress

Next, we tested the hypothesis that inhibition of autophagy, and accompanying increases in the abundance of ROF1 and HSP90 during HS recovery impact the nuclear stability of HSFA2. In accordance with the hypothesis, treatment with 3-methyladenine (3-MA) resulted in higher nuclear accumulation of HSFA2-YFP in *pHSFA2:HSFA2-YFP* seedlings than in DMSO-treated controls (Figure 6C,D). Notably, we did not detect HSFA2-YFP as vacuolar puncta resembling autophagic bodies following treatment with the autophagy inhibitor with ConA (Figure S6B,C and Video S8). Furthermore, HSFA2-GFP did not co-localize with RFP-ATG8b-labeled autophagosomes (Figure S6C). Together, we concluded that HSFA2 is not an autophagy target (Figure S6). Collectively, our results strongly suggest that the higher levels of ROF1 and HSP90 proteins in *NBR1-*deficient plants enhance expression of HSFA2-regulated genes during the HS recovery phase, thereby preparing *Arabidopsis* for an improved response to the next HS (Figure 7).

## Discussion

Multiple recent lines of evidence showed that PQC plays an important role in the regulation of HS memory and recovery. A key process in PQC is autophagy, which has recently been reported to be involved in the regulation of HS memory, *via* mechanisms that have not yet been elucidated in detail [51,52]. In this study, we uncovered an essential function of NBR1, an autophagy receptor protein, in regulating plant responses to repeated HS. We present several lines of evidence demonstrating that *Arabidopsis* NBR1 selectively targets and coordinates the autophagic degradation of a central HS memory control module consisting of HSP90.1 and ROF1, thereby hastening recovery from HS but weakening responses to future heat stress.

Using confocal microscopy, we detected an increased accumulation of NBR1-containing bodies during the HS recovery phase in *Arabidopsis*, which resemble autophagic bodies (Figure 1). NBR1 itself is a substrate for autophagy and is recycled along with its cargo during the process of selective clearance [30,41,46,53]. Higher accumulation of NBR1 protein in autophagy-deficient mutants (compared to WT plants) confirmed that NBR1 is an autophagy substrate, and possibly an autophagy receptor, during the recovery from HS (Figure 2). Additionally, co-localization of ATG8b-containing autophagosomes with the NBR1 bodies, and co-immunoprecipitation of ATG8a with NBR1 (Figure 2), demonstrated a functional association between NBR1 and autophagy during the HS recovery phase.

To examine the functional involvement of NBR1 in HS recovery the response to repeated HS, phenotypes of *NBR1*-loss-of function mutant (*nbr1-2*) plants were assessed following exposure to different HS regimes. *nbr1-2* seedlings were impaired in basal HS tolerance when plants were subjected to a single severe HS, in comparison to WT plants (Figure S1), in agreement with previous reports [38,42]. In contrast, *nbr1-2* mutants had higher survival rates following severe HS (4 d) after a non-lethal (priming) HS treatment (Figure 1). Increasing experimental evidence indicates that a plant's respons to single and recurrent incidences of HS differ, suggesting the involvement of distinct (but possibly overlapping) genetic/regulatory pathways [10,11,15,20,25]. Phenotypically, the *nbr1-2* mutant resembles autophagy mutants [25], suggesting participation of NBR1 in cellular recovery after release from heat stress, and thus in weakening memory of HS.

Although a role for NBR1 as a selective autophagy cargo receptor was identified several years ago, its substrates have remained largely unknown. Consistent with the presence of ubiquitin-binding UBA domain, NBR1 has been connected with the clearance of ubiquitinated protein aggregates derived from misfolded proteins that accumulate under stress, thereby directing their autophagic turnover in plants [42,43,46,54]. To define the importance of NBR1 in regulating HS recovery, we first identified potential NBR1 cargo by two comprehensive proteomic approaches, either involving a comparative LC-MS/MS analysis of *nbr1-2* and WT plants or a LC-MS/MS analysis of proteins that bind NBR1-GFP versus a GFP control. When the lists were aligned, 58 high-confidence NBR1 interactors were identified, which notably included a number of proteins connected to translation, metabolism, protein chaperones and proteolysis, consistent with a role for NBR1 in promoting PQC. To our knowledge, this is the first attempt to identify in bulk *Arabidopsis* proteins that interact with NBR1 *in vivo* and are potentially recruited by this receptor during autophagy.

Among the identified potential cargo, we highlighted HSP90 and ROF1 with previously known roles in regulating HS memory [22,45]. Co-localization analysis, BiFC and immunoblotting were used to further validate the association of NBR1 with HSP90.1 (a stress-inducible cytosolic isoform of HSP90) and ROF1 (Figure 4). Of note, we showed that deletion of the UBA sequences in NBR1 did not impair the interaction between NBR1 and ROF1 or HSP90.1, implying that the association of NBR1 with either ROF1 or HSP90.1 is independent of ubiquitination and the ubiquitin/26S proteasome system. In accordance with this, recent studies demonstrated that NBR1 can sometimes interact with its cargo in an ubiquitin-independent manner [30,45,55]. 

Selective turnover of HSP90 by autophagy has been recently reported, but the receptor mediating recruitment of HSP90 to the autophagosomes remained unknown [25]. In this study, we show by comprehensive cellular, biochemical and molecular analyses that ROF1 and HSP90 are degraded by NBR1-mediated autophagy during the HS recovery phase. Moreover, weshowthat ROF1 and HSP90 significantly accumulate in the vacuole of WT plants upon treatment with autophagy inhibitors, whereas accumulation of both proteins is dramatically reduced in *nbr1-2* mutant plants (Figure S3). Collectively, our data provide compelling evidence for a role of NBR1 in delivering ROF1 and HSP90.1 to the vacuole *via* the autophagy machinery during the HS recovery phase.

Plant responses to HS involve finely tuned interaction networks, which, *inter alia*, engage HSF transcription factors, HSPs, and their co-chaperones [46,56]. HSP90s are among the most important molecular chaperones in eukaryotic cells. In concert with cognate co-chaperone molecules, they play important roles in numerous essential cellular processes, including signal transduction, protein targeting, and stress protection [57–59]. ROF1 is a plant homolog of FK506-binding proteins such as FKBP4/FKBP52, which act as co-chaperones within the HSP90 machinery [58]. Based on prior binding studies, *Arabidopsis* ROF1 specifically interacts with the HSP90.1 isoform, and upon exposure to HS, HSP90.1 also directly interacts with HSFA2, the only HSF (out of 21) with a specific function in HS memory and responses to repeated HS [20,60,61], leading to the formation and nuclear import of ROF1-HSP90.1-HSFA2 complexes.

In this study, we report a novel role for NBR1 in counterbalancing the impact of the HSP90.1-ROF1 complex during the HS recovery through directed autophagic turnover of the complex (Figure 7). Consistent with this activity, we detected significantly higher expression of *HSFA2* and its target genes in *nbr1-2* mutants versus WT plants (Figure 6). Higher transcription of *HSFA2* is consistent with a recent demonstration of HSFA2 self-regulating *via* a feedforward loop involving the H3K27me3 demethylase/REF6 (RELATIVE OF EARLY FLOWERING 6) after exposure to a moderate HS [62]. This turnover would suppress the activation of HSFA2 and thus weaken memory of the prior HS. Although HSFA2 does not appear to be an autophagy substrate based on our localization studies (Figure S6), inhibition of autophagy with 3-MA did cause the hyperaccumulation of HSFA2-YFP in the nucleus (Figure 6), suggesting a link between ROF1-HSP90.1-HSFA2 complexes and the nuclear retention of HSFA2.

Based on these findings, we propose a model whereby HSP90.1 and ROF1 associate in the cytoplasm during HS recovery, and upon further interaction with HSFA2 are imported to the nucleus [22] (Figure 7). The presence of the ROF1-HSP90-HSFA2 complex in the nucleus triggers enhanced expression of HSFA2-regulated genes, ultimately required for a robust protective response to recurrent HS [22,63]. NBR1 deactivates this protection by binding to cytoplasmic HSP90-ROF1 and directing its transport to autophagic vesicles for turnover. Clearly, further clarification for how NBR1 recognizes HSP90.1-ROF1 as a cargo, which we propose is independent of ubiquitination, should help unravel this effect. Intriguingly, we identified numerous ribosomal subunits as NBR1 interactors, suggesting that NBR1 also functions as a receptor for selective autophagy of ribosomes (ribophagy) during recovery from HS in *Arabidopsis*. While ribophagy has been explored extensively in other organisms, including yeast and mammalian cells [64,65], and has been inferred by studies on rRNA turnover [66] with *Arabidopsis* and proteomic studies with maize [67], its mechanism(s) remain unclear in plants. Certainly, ribosome turnover during normal and stress conditions is crucial for optimal PQC and might also be linked to the HS memory machinery.

In summary, we show here that NBR1-mediated selective autophagy plays a major role in a plants’ responses to repeated HS, through its control of HSP90.1 and ROF1 levels, thereby enhancing our understanding of fundamental plant physiology and indicating new strategies for improving their performance in rapidly changing environments.

## Materials and methods

### General

All oligonucleotides used in the study (Table S2) were obtained from Eurofins, MWG Operon (Ebersberg, Germany).

### Plant materials and growth conditions

*Arabidopsis thaliana* ecotype Col-0 was used as wild-type for all experiments. Seeds of wild type and the mutant and transgenic lines *nbr1-2* [30], *pNBR1:NBR1-GFP* [30], *35S:YFP-mCHERRY-NBR1* [46], *35S:TUB-RFP* [68], *atg5-1* [44], *atg18a-2* [25], *rof1-2* (*WiscDS_LOX_D502D06*) were surface-sterilized and sown in Petri dishes containing Murashige-Skoog (MS) (Duchefa, M0222.0050) agar (Duchefa, M10025000) medium supplemented with 1% (w:v) sucrose (Sigma-Aldrich, S0389). They were stratified at 4°C in darkness for 2 d, and the seedlings were grown under a diurnal cycle of 16 h light (120 µE m^−2^ s^−1^) at 22°C and 8 h dark at 22°C.

### Plasmid construction and generation of transgenic lines

*ROF1* and *HSP90.1* coding sequences without stop codon were amplified by PCR from *A. thaliana* heat-induced seedling cDNA (using primers listed in Table S2) and cloned into *pDONR207*(ABRC, V1008805106) using BP clonase (Thermo Fisher Scientific, 11789020). The sequence-verified entry vector was recombined into *pUBC-DEST-mRFP* [69] to generate *pUBQ10:ROF1-RFP* and *pUBQ10:HSP90.1-RFP* by LR recombination using LR reaction mix II (Thermo Fisher Scientific, 11791020). GATEWAY entry vectors with *NBR1* and *NBR1-∆UBA* (deletion of the C terminal ubiquitin binding domains) [46] coding sequences without stop codon were recombined into *pUBQ10-DEST-eGFP* [69] to generate *pUBQ10:NBR1-GFP, pUBQ10:NBR1-∆UBA-GFP* by LR recombination using LR reaction mix II (Thermo Fisher Scientific, 11791020). The *ATG8b* coding sequence contained in plasmid pENTR223 (ABRC, G22921) was recombined into *pUBN-mRFP-DEST* [69] to generate *pUBQ10:RFP-ATG8b*.

The *pHSFA2:HSFA2-GFP* construct was generated by GATEWAY cloning. First, the CaMV *35S* promoter was removed from pK7FWG2.0 using *Spe*I and *Stu*I restriction sites. Then a multi-cloning site (MCS) encompassing *Spe*I, *Nru*I, *Bgl*II, *Xho*I, and *Stu*I restriction sites was inserted using primers pK7FWG2_MCS Fwd and Rev (Table S2) and the NEBuilder® HiFi DNA Assembly (New England Biolabs, E2621L) to allow insertion of other promoter fragments for gene expression analyses. The resulting vector was named pK7FWG2_wo35S. Next, the promoter fragment of *HSFA2* (~1.2 kb) was amplified by PCR from wild-type genomic DNA (*pHSFA2* Fwd and Rev primers, Table S2). *Xho*I and *Spe*I cutting sites were added to the ends of the promoter fragment by PCR. Afterward, the sequence-verified PCR fragment was cloned into pK7FWG2_wo35S *via* restriction and ligation resulting in pK7FWG2_*promHSFA2*. Subsequently, *HSFA2 (At2g26150)* genomic DNA (with no stop codon) was amplified by PCR from wild-type genomic DNA (*HSFA2* Fwd and Rev primers, Table S2). The sequence-verified PCR product was cloned into pDONR201 using BP clonase. The sequence-verified entry vector was recombined into pK7FWG2_*promHSFA2* to generate pK7FWG2_*promHSFA2_HSFA2* (*pHSFA2:HSFA2-GFP*) by LR recombination using LR reaction mix II (Thermo Fisher Scientific, 11791020). The recombined destination vector *pHSFA2:HSFA2-GFP* was electroporated into *Agrobacterium tumefaciens* strain GV3101.

For BiFC analysis, all constructs were generated *via* the 2in1-cloning system as described [69]. Briefly, full-length coding fragments of *HSP90.1, ROF1, NBR1*, and *NBR1-∆UBA* were amplified by PCR and cloned into 2in1 entry vectors (*pDONR*™*221 P2P3* for *HSP90.1, ROF1*, and *pDONR221*™-*P1P4* for *NBR1* and *NBR1-∆UBA*) using BP clonase. The sequence-verified entry vectors recombined into 2-in-1 destination vector (*pBiFC-2in1* vectors) [69] following LR recombination. The NBR1 coding sequence was tagged at its 5‘ end with that for *nYFP*, while *ROF1, HSP90.1* were tagged at its 3ʹ end with that for *cYFP*. Primers used in the study are listed in Table S2. The recombined destination plant vectors were electroporated into *Agrobacterium tumefaciens* strain GV3101 and transformed into *Arabidopsis* wild type, *pNBR1:NBR1-GFP, pACT2:GFP-ATG8a* [25], *nbr1-2 atg5-1* [70], and *nbr1-2* plants by the floral dip method [71].

### Heat stress treatments of Arabidopsis thaliana seedlings

The priming experiments were performed with seedlings in Petri dishes as previously described [10,11]. Briefly, for the priming (mild heat) stress treatment, 5-d-old seedlings were exposed to 37°C for 1.5 h in an incubator, then at 22°C for a 1.5 h recovery period, followed by 45 min of heat stress at 44°C (in a hot water bath). After the priming treatment, seedlings were transferred to normal growth conditions (16-h light/8-h dark photoperiods) at 22°C for 4 d (designated the HS recovery phase), during which samples were harvested for analyses.

### Chemical inhibitor treatments

Chemical inhibitor treatments were performed as described [25]. Six‐day‐old primed seedlings were transferred to 3 mM MES buffer containing 1 µM ConA (Sigma-Aldrich, 80890–47-7) and 5 mM 3-MA (Sigma-Aldrich, SAE0107) dissolved in dimethyl sulfoxide (DMSO) or 0.1% [v:v] DMSO (Sigma-Aldrich, D5879) alone as control treatment for 6 h and subjected for subsequent microscopy analysis. For immunoblotting, we transferred 6-d-old-primed seedlings to liquid culture medium (MS medium supplemented with 1% [w:v] sucrose) containing 1 µM ConA dissolved in DMSO or DMSO alone (control treatment) for 12 h in the dark. The seedlings were harvested at indicated time points after the treatment and total proteins were analyzed by immunoblotting.

### Sample collection, total protein extraction and in-solution trypsin digestion for LC-MS/MS analysis

Five-day-old Col-0 and *NBR1*-deficient (*nbr1-2*) *Arabidopsis* seedlings were harvested during the HS recovery phase (2 d after priming). Controls with no heat priming treatment were harvested at the same time point. The samples were pulverized at liquid nitrogen temperatures and then subjected to phase separation and total protein extraction as described [72]. To extract proteins, 100 mg portions of ground tissue were suspended in methyl tertiary butyl ether (MTBE) buffer, then the suspensions were incubated at 4°C for 15 min, sonicated, and centrifuged at 20,000 *g* at 4°C. Proteins in the resulting pellet were dissolved in buffer containing 6 M urea (Roth, 2317.1), 2 M thiourea (Sigma-Aldrich, 88810–500 G), 15 mM DTT (Sigma-Aldrich, D0632-25 G), and protease inhibitors (Sigma-Aldrich, 4693159001)), and clarified by centrifugation at 10,000 *g* for 5 min. Concentrations of solubilized proteins in the supernatants were quantified by Bradford analysis. For proteomic analysis, portions of extracts containing 50 µg of protein were digested either in-solution or by FASP column digestion [73] using a trypsin/Lys-C mixture (Mass Spec Grade; Promega, V5073) according to the manufacturer’s instructions. Digested peptides were desalted on C18 SEP-Pak columns (Teknokroma, TR-F034000), which were attached to a QIAvac 24 Plus (Qiagen, Hilden, Germany) vacuum manifold, then analyzed by LC-MS/MS using a Q Exactive HF high-resolution mass spectrometer coupled to an ACQUITY UPLC M-Class System (Waters, Milford, USA). Raw data were processed using MaxQuant software [74] using the *Arabidopsis thaliana* TAIR10 protein annotation (Arabidopsis TAIR database Version 10, The Arabidopsis Information Resource, www.Arabidopsis.org) in combination with the search engine Andromeda [75].

### Transient expression experiments

All colocalization and BiFC assays were performed by transiently expressing constructs introduced into *Nicotiana benthamiana* plants by *Agrobacterium* infiltration, as described [76]. Leaf sections of infiltrated plants exposed to mild heat stress and non-stressed controls were subjected to confocal microscopy analysis 2 d after infiltration, as previously described [25].

### Immunoblot analysis

Total protein extraction, fractionation, and immunoblotting were done as described [11,25,36,52]. Briefly, proteins were separated on denaturing 12% polyacrylamide gels and electroblotted onto nitrocellulose membrane (GE Healthcare, 10600001). Proteins of interest were then detected using a mouse monoclonal antibody against RFP (Chromotek, 6G6; 1:1,000), and rabbit polyclonal antibodies against NBR1 (Agrisera, AS142805; 1:2,000), HSP90.1 (Agrisera, AS08346; 1:3,000), GFP (Invitrogen, A11122; 1:1000), histone H3 (Abcam, ab1791; 1:5,000) and FBP/FBPase (Agrisera, AS04043; 1:5000) as the primary antibodies. IRDye 800CW-conjugated goat anti-rabbit IgG (H + L) and goat anti-mouse IgG (H + L) (LI-COR Biosciences; 926–32,211) antibodies were used for detection at 1:10,000 dilutions. Images of immunoblots were captured using the Odyssey Infrared Imaging System (LI-COR Biosciences).

Immunoblots were analyzed following the manufacturer´s (LI-COR Biosciences) instructions. Care was taken not to saturate the signals obtained from the scanners, and the blots were semi-quantitatively analyzed using ImageJ software (https://imagej.nih.gov/ij/) by comparing the protein band of interest with the respective loading control as described [77]. Briefly, scanned immunoblot membranes were analyzed by ImageJ after employing the background subtraction using the rolling ball radius method. Subsequently, each band was individually selected and bounded with rectangular box-type region of interest (ROI) selection and the ´Gels´ function. Next, peak areas were quantified and data acquired as arbitrary values.

### Expression profiling by qRT-PCR

Total RNA extraction, cDNA synthesis, and qRT-PCR were performed as described [78]. All genes included in the qRT-PCR experiments are listed in Table S2. qRT-PCR primers, designed using QuantPrime (www.quantprime.de) [79] and the ABI-PRISM 7900 HT sequence detection system (Applied Biosystems, Darmstadt, Germany), was used for the PCR amplifications and products were visualized using SYBR Green (Life Technologies, 4368706). *ACT2/ACTIN2* (*AT3G18780*) served as a reference gene for data analysis.

### In planta co-immunoprecipitation (CoIP) analysis

CoIP assays were performed as described [25] with minor modifications. Five-day-old *35S:GFP, 35S:GFP-ATG8a, pNBR1:NBR1-GFP* and *pACT:GFP-ATG8a/pUBQ10:ROF1-RFP* seedlings were subjected to priming stimulus and samples were cross-linked and harvested at the indicated time points into the HS recovery phase. *pNBR1:NBR1-GFP* and *35S:GFP* seedlings were homogenized in liquid nitrogen and total proteins were extracted with extraction buffer containing 50 mM Tris-HCl (pH 7.5), 150 mM NaCl, 1% Tween-20 (Sigma-Aldrich, P1379) and 1/2 a tablet of protease inhibitor cocktail (Sigma-Aldrich, 11,836,145,001). The supernatant was filtered through a 0.2-μm filter (Whatman) after centrifugation. Total proteins were incubated with anti-GFP antibody-decorated microbeads (Miltenyi Biotec, Germany) overnight at 4°C on a rotary shaker. Washing steps were performed by following the manufacturer’s instruction. The proteins were eluted using 8 M urea buffer to release the immunoprecipitated proteins. A fraction of eluted proteins were digested and cleaned for LC-MS analysis as described [71].

The same protein extraction protocol was used for *35S:GFP-ATG8a, 35S:GFP, pACT2:GFP-ATG8a/pUBQ10:ROF1-RFP* and *35S:TUB-RFP* seedlings with slight modification, as mentioned below. Five-day-old seedlings were subjected to priming stimulus and samples were cross-linked and harvested 2 d into the HS recovery phase as reported [25]. The harvested seedlings were homogenized in liquid nitrogen and total proteins were extracted in 50 mM Tris-HCl, pH 7.5, 150 mM NaCl, 2% Tween-20 (Bio-Rad, 1706531), 1/2 tablet cocktail protease inhibitor (Sigma-Aldrich, 11836145001). The mixture was clarified at 20,000 *g* for 20 min at 4°C, and the resulting supernatant was filtered through a 0.2-μm filter (GE Healthcare, 10462200). Total proteins were incubated with anti-GFP antibody microbeads (Miltenyi Biotec, 130–091-125) for GFP-ATG8a and GFP and anti-RFP antibody beads (Chromotek, rtma-20) for ROF1-RFP and TUB-RFP for 6 h at 4°C on arotary shaker. The beads were washed following the manufacturer’s instruction, then bound proteins were eluted using buffer containing 50 mM Tris HCl, pH 6.8, 50 mM DTT, 1% SDS, 1 mM EDTA, 0.005% bromophenol blue, and 10% glycerol (Sigma-Aldrich, G9012). The eluted proteins were immunoblotted using polyclonal antisera recognizing proteins of interest, as mentioned in the text.

### Microscopy analysis

Fluorescence signals were imaged using a confocal scanning microscope (Leica TCS-SP8). The manufacturer’s instructions were followed for settings used to collect the fluorescent signals from chlorophyll (auto-fluorescence) and the eGFP, mRFP, and eYFP reporters. Line sequential scanning mode with dual-channel observation was applied to avoid possible bleed-through of signals from two fluorophores. All images were processed by using ImageJ software (https://imagej.net/Fiji). To distinguish between vacuolar and cytoplasmic localization of proteins, we performed time-series analyses. We then chose a representative optical section for presentation of the data in the manuscript.

### Statistical analysis

The significance of differences between means was assessed used Student’s *t-*test, and differences were deemed significant if *p ≤ 0.05*.

## Supplementary Material

Supplemental Material

## Data Availability

The mass spectrometry proteomics data were deposited in the ProteomeXchange Consortium via PRIDE [80] partner repository with the dataset identifier PXD015027.
