# Supplementary material for: Selective autophagy regulates heat stress memory in Arabidopsis by NBR1-mediated targeting of HSP90.1 and ROF1
Source: Autophagy. 2020 Sep 24;17(9):2184–99. doi: 10.1080/15548627.2020.1820778 (PMC8496721; doi:10.1080/15548627.2020.1820778)
Supplement: Supplemental Material [file KAUP_A_1820778_SM4625.zip › Supplementary information/Supplementary Figures-Thirumalaikumar et al.-SB R3.docx]

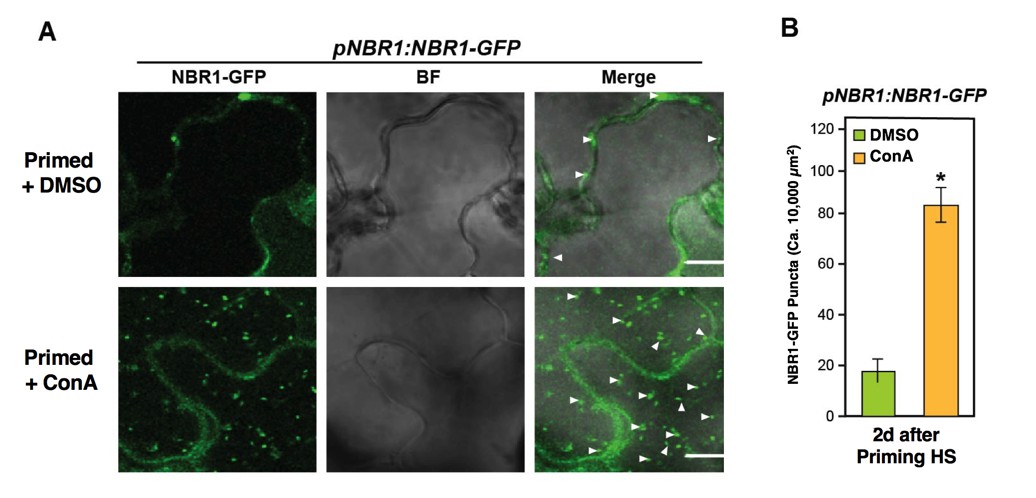


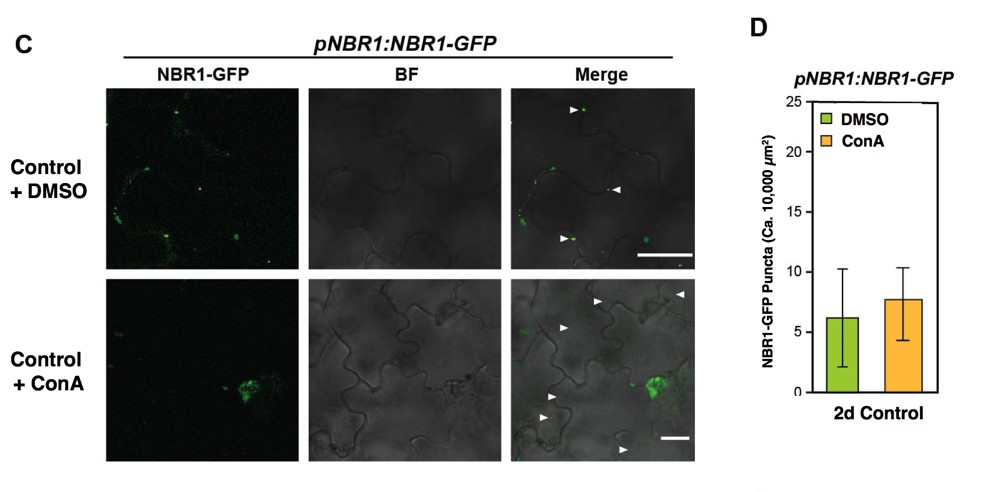


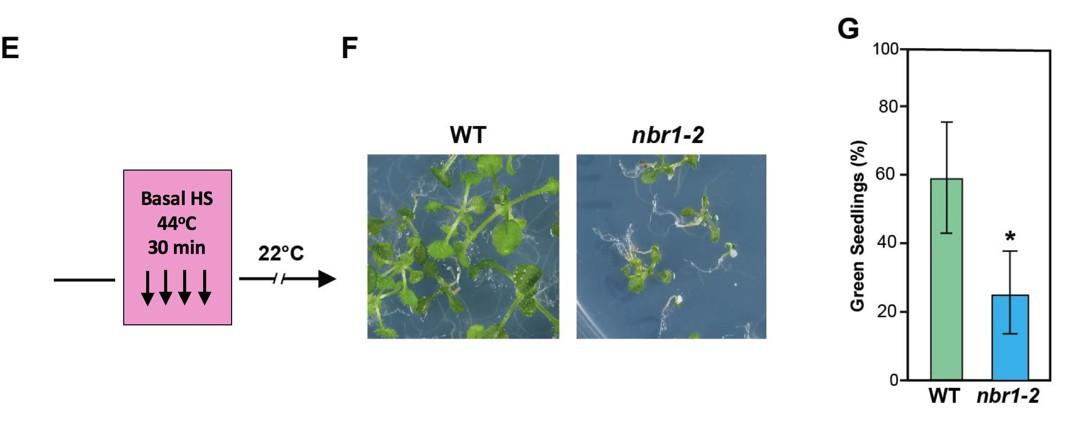


**Figure S1.** NBR1 is degraded in the vacuole during the HS recovery phase and NBR1-deficient mutants are sensitive to heat stress. (**A**) NBR1-GFP puncta are imported into the central vacuole by autophagy during the HS recovery phase. Accumulation of NBR1-GFP puncta in *pNBR1:NBR1-GFP* seedlings 2 d after priming HS treatment assessed in cotyledons by fluorescence confocal microscopy following DMSO control and ConA (1 µM) treatment. BF, bright field. Arrowheads indicate NBR1 bodies. Scale bars: 25 µm. (**B**) Frequencies of NBR1-GFP puncta 2 d after HS priming with and without ConA treatment per 10,000 µm^2^ area of leaf epidermis section. Data are means ± SD (n=6). Asterisks indicate significant (*p* < 0.05) differences between samples of plants subjected to ConA and DMSO control treatment according to Student’s *t*-test. (**C**) NBR1-GFP puncta are imported into the central vacuole by autophagy under control (unprimed) condition. Accumulation of NBR1-GFP puncta in *pNBR1:NBR1-GFP* seedlings at control condition (2 d) assessed in cotyledons by fluorescence confocal microscopy following DMSO control and ConA (1 µM) treatment. BF, bright field. Arrowheads indicate NBR1 bodies. Scale bars: 25 µm. (**D**) Frequencies of NBR1-GFP puncta under control condition with and without ConA treatment per 10,000 µm^2^ area of leaf epidermis section. NS, not significant. Data are means ± SD (n=6). Asterisks indicate significant (*p* < 0.05) differences between samples of plants subjected to ConA and DMSO control treatment according to Student’s *t*-test. (**E**) Scheme of heat stress (HS) regime for analysis of basal HS. Seven-day-old *nbr1-2* (NBR1-deficient mutant plants) and Col-0 wild-type (WT) seedlings were subjected to HS at 44**°**C for 30 min. Following HS, seedlings were incubated in normal growth conditions for 14 d, during which the seedlings were photographed. (**F**) Basal heat stress tolerance phenotypes of *nbr1-2* and Col-0 WT seedlings. Bleaching indicates susceptibility to HS. (**G**) Percentages of green seedlings (indicative of seedling survival rates) are shown in the bar graph. Data are means ± SD (n=4). Asterisks indicate significant (*p* < 0.05) differences between *nbr1-2* and WT plants.


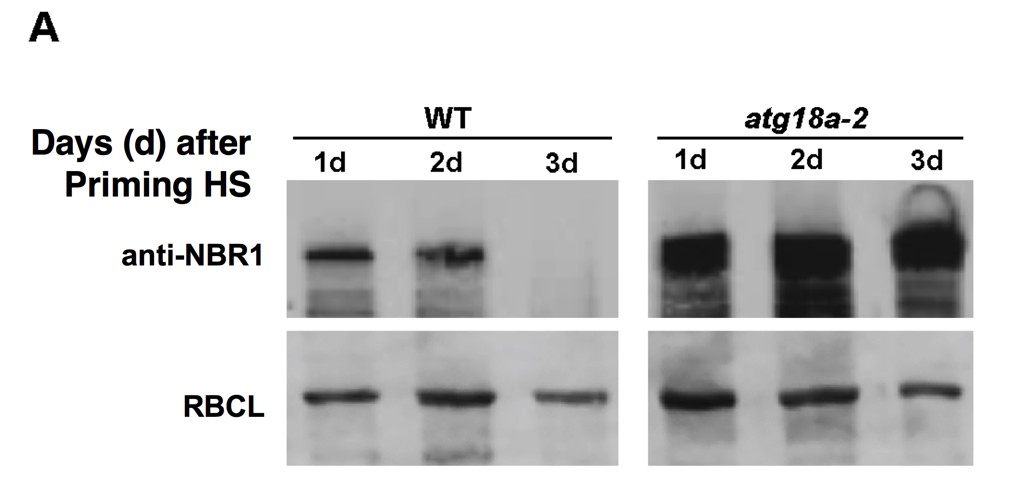


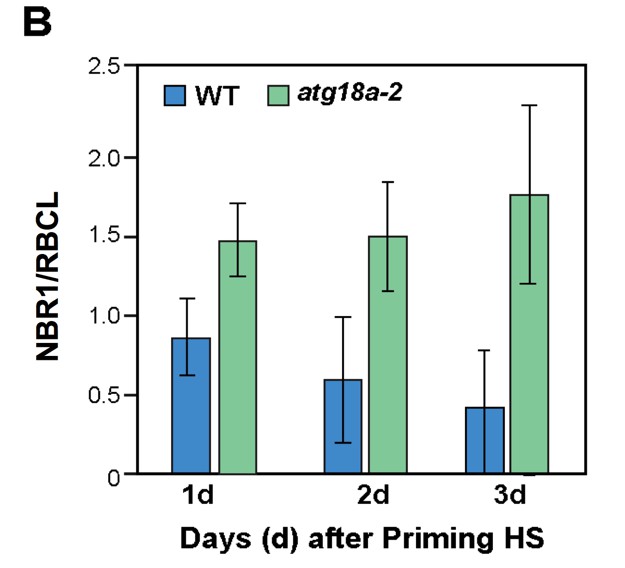


**NBR1:RBCL**

**Figure S2.** Accumulation of NBR1 in *atg18a-2* deficient plants. (**A**) Immunodetection of NBR1 during the HS recovery phase in *atg18a-2* and wild-type (Col-0) seedlings using anti-NBR1 antibody (Agrisera, AS142805). Ponceau-stained RBCL was used as a loading control (bottom panels). Relative intensities (NBR1:RBCL) are shown as numerical values. (**B**) Signals of immunoblot analyses were quantified using ImageJ and normalized to the amount of RBCL in the same samples. Means and standard deviation are given (n=3, independent biological replicates).


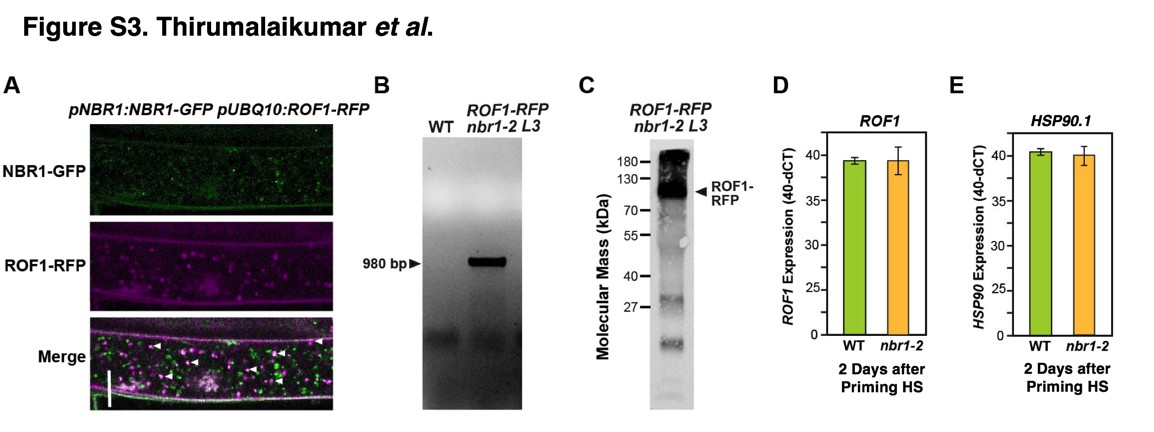


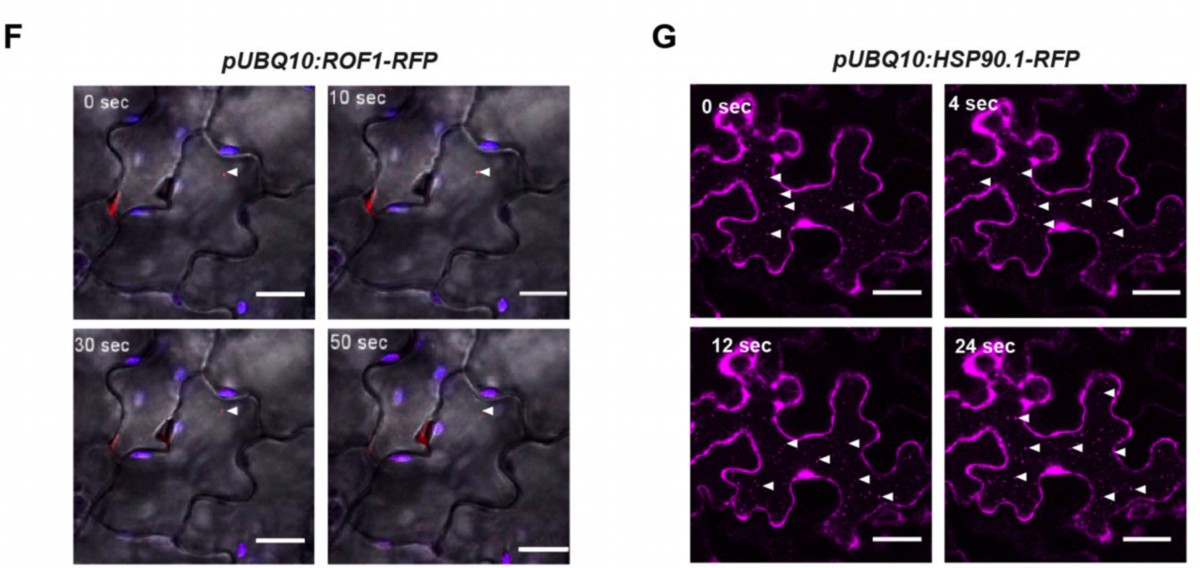


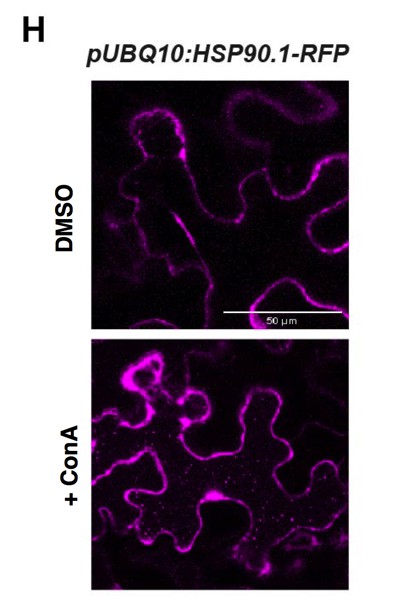


***pUBQ10:ROF1-RFP***

***pUBQ10:HSP90.1-RFP***

***pUBQ10:HSP90.1-RFP***

**DMSO**

**+ConA**


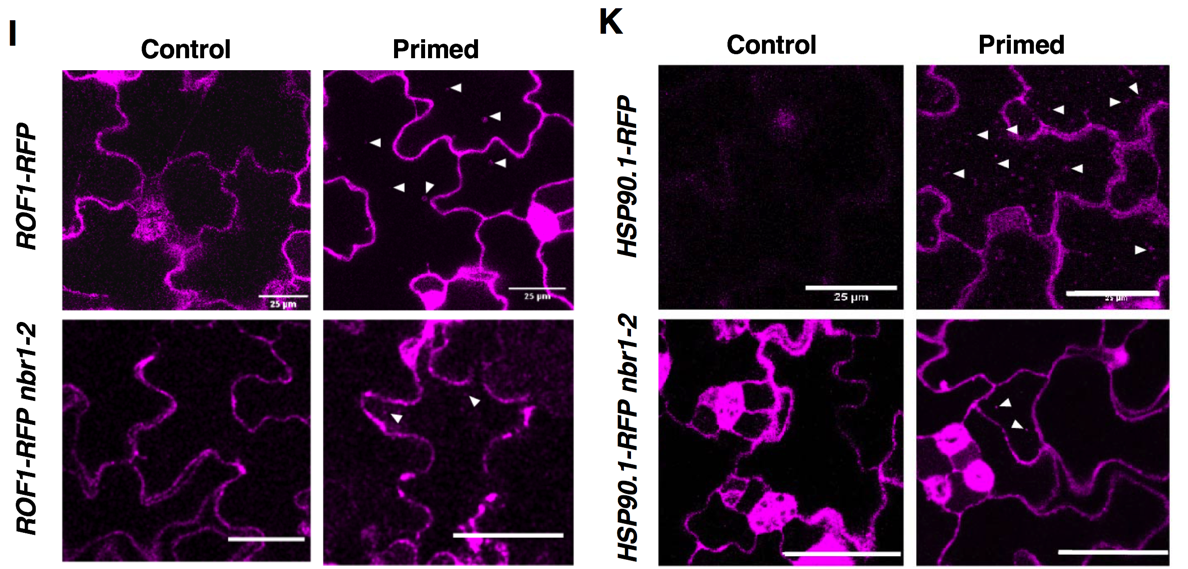


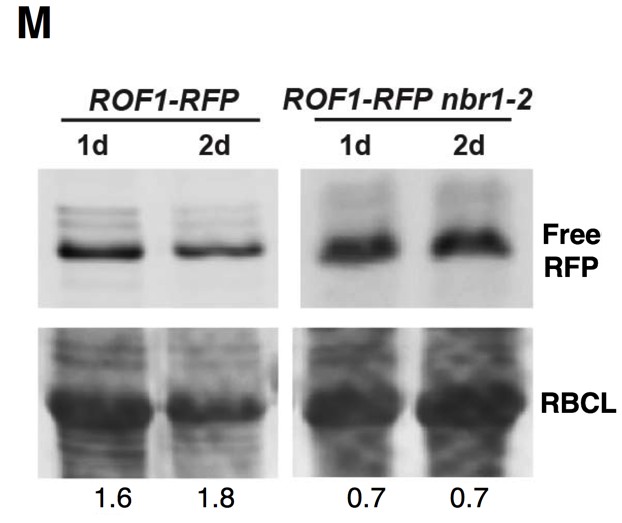


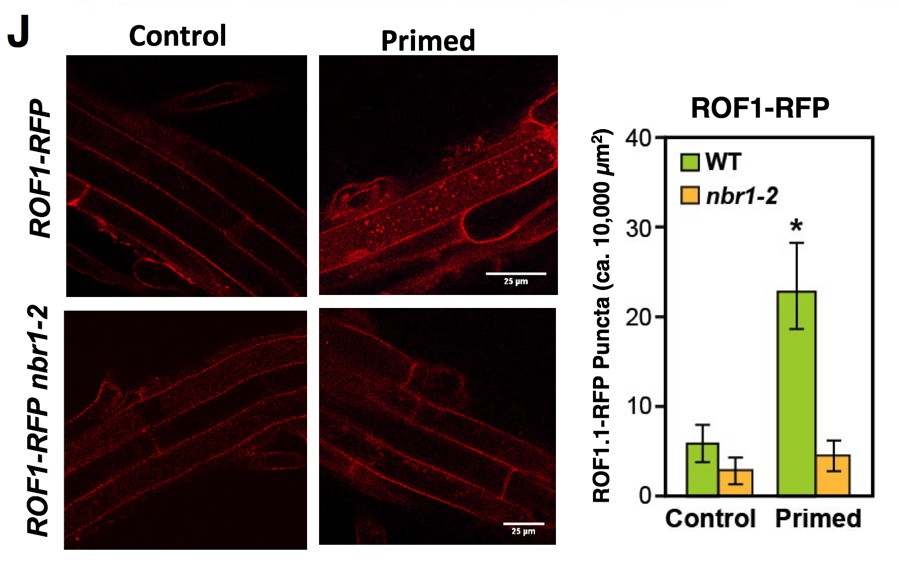


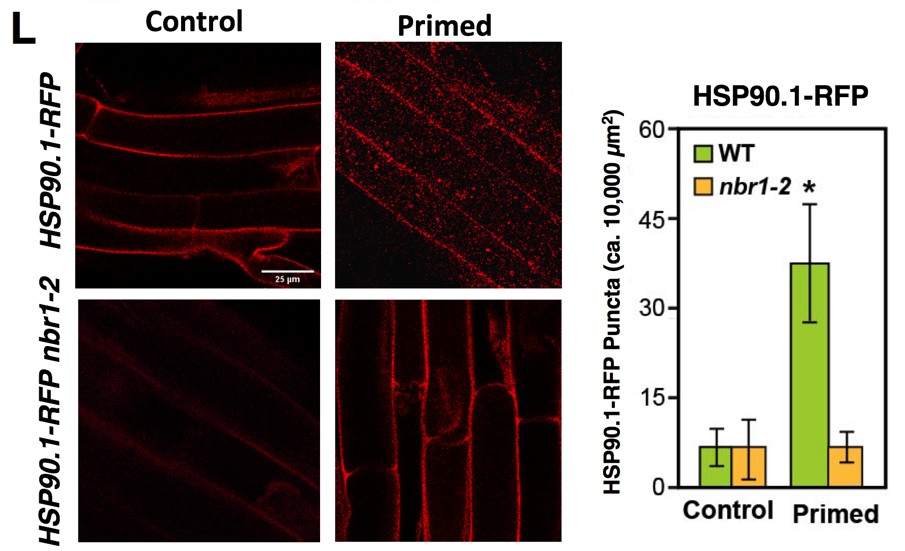


**Figure S3.** NBR1 mediates ROF1 and HSP90 degradation by the autophagy pathway. (**A**) Colocalization of NBR1-GFP/ROF1-RFP in root tissues. Seedlings were assessed by fluorescence confocal microscopy during the HS recovery phase (2 d after priming HS) following ConA treatment. The white puncta (green + magenta) represent colocalization. Scale bars: 25 µm. (**B**) Validation of *ROF1-RFP/nbr1-2* plants. Transcript levels of *ROF1-RFP* were detected by end-point PCR (left panel). **(C)** ROF1-RFP protein was detected by anti-RFP antibody (Chromotek, 6G6; 1:1,000), seedlings were harvested 2 d into the HS recovery phase (right panel). M denotes the marker. (**D**) and (**E**) Expression levels of *ROF1* and *HSP90.1* in Col-0 WT and *nbr1-2* mutant seedlings during the HS recovery phase (2 d), determined by qRT‐PCR. Values are differences between an arbitrary value of 40 and dCt, calculated as described [11], means ± SD (n = 3, where n represents independently performed experiments). (**F**) Time-lapse images of ROF1-RFP in Col-0 background during HS recovery phase. *pUBQ10:ROF1-RFP* seedlings were subjected to priming HS and 2 d later cotyledons were subjected to ConA treatment and visualized under a confocal microscope**.** Scale bars: 25 µm. (**G**) Time-lapse imaging of HSP90-RFP. The *pUBQ10:HSP90-RFP* construct was infiltrated into *Nicotiana benthamiana*, and two days later leaf sections were treated with ConA and assessed by fluorescence confocal microscopy. Note: Image present in F is the time scale representation from the panel H, lower panel. Scale bars: 25 µm. **(H)** HSP90.1-RFP is imported into the central vacuole by autophagy. *pUBQ10:HSP90-RFP* was infiltrated into *N. benthamiana* and 2 d later leaf sections were treated with DMSO control and ConA before they were assessed by confocal fluorescence microscopy. Scale bars: 25 µm. (**I**) Confocal fluorescence microscopy analysis of ROF1-RFP revealed impairment in delivery of ROF1-RFP to the central vacuole in *nbr1-2* mutants. *nbr1-2* seedlings were subjected to priming HS. Two days after priming, HS cotyledons were subjected to ConA treatment and visualized under a confocal microscope. Unprimed (control) seedlings (treated with ConA) were used as control. Arrowheads show ROF1-RFP in the central vacuole. Scale bars: 25 µm. (**J**) Left panel, confocal analysis of ROF1-RFP in root tissues revealed impairment in delivery of ROF1-RFP to the vacuole in *nbr1-2* mutants. *ROF1-RFP* and *ROF1-RFP/nbr1-2* seedlings were subjected to priming HS. Two days after roots were subjected to ConA treatment and visualized under a confocal microscope. Right panel, frequencies of ROF1-RFP puncta under control (unprimed) condition and 2 d after HS priming with ConA treatment per 10,000 µm^2^. Data are means ± SD (n=6). Scale bars: 25 µm. (**K**) Confocal fluorescence microscopy analysis revealed a remarkable reduction in vacuolar delivery of HSP90.1-RFP in *nbr1-2* compared to Col-0 plants after priming treatment (2 d). Unprimed (control) seedlings (treated with ConA) were used as control. Arrowheads show HSP90.1-RFP in the central vacuole. Scale bars: 25 µm. (**L**) Left panel, confocal fluorescence microscopy analysis HSP90.1-RFP in root tissues revealed a significant reduction in vacuolar delivery of HSP90-RFP to the vacuole in *nbr1-2* compared to Col-0 plants after priming treatment (2 d). Right panel, frequencies of HSP90.1-RFP puncta under control (unprimed) condition and 2 d after HS priming with ConA treatment per 10,000 µm^2^. Data are means ± SD (n=6). Asterisks in panels I and J indicate significant (*p* < 0.05) differences between Col-0 (WT) and *nbr1-2* mutants according to Student’s *t*-test. Scale bars: 25 µm. (**M**) Free RFP analysis of *ROF1-RFP* and *ROF1-RFP/nbr1-2* plants during the HS recovery phase by immunodetection using an anti-RFP antibody (upper panels). Ponceau-stained RBCL was used as a loading control (bottom panel). Note, the free RFP level is higher in Col-0 plants than in *nbr1-2* plants, indicating impaired delivery of ROF1 to the vacuole in the latter.


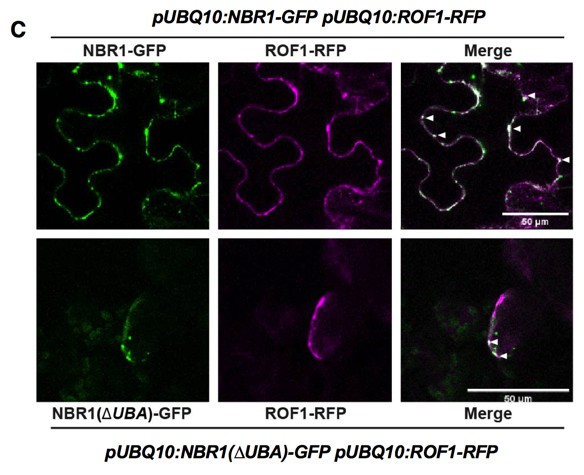


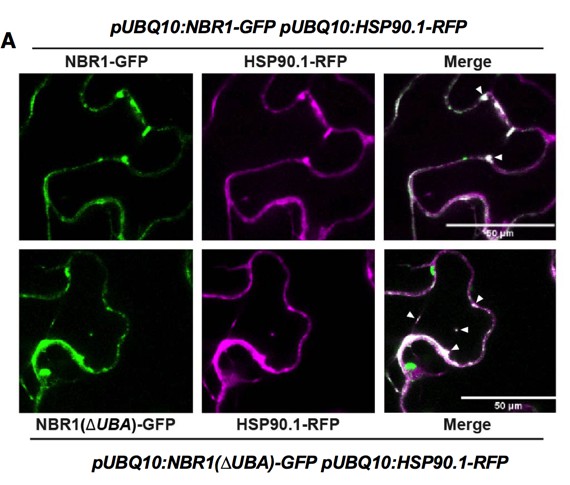


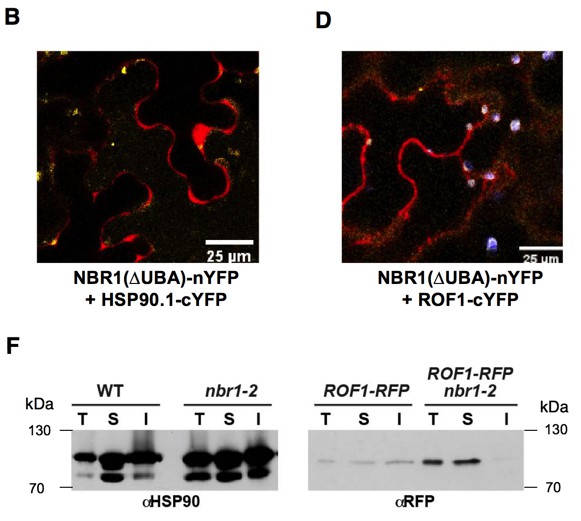


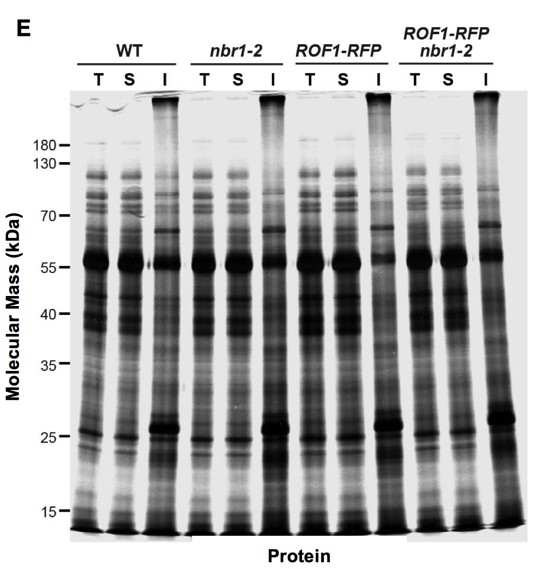


**anti-RFP**

**anti-HSP90**

**Figure S4.** NBR1 targeting of HSP90.1 and ROF1 is independent of ubiquitination. (**A**) Colocalization of NBR1-GFP and NBR1-∆UBA-GFP (deletion of ubiquitin-binding domains) with HSP90.1-RFP. Imaging was done 2 d after *A. tumefaciens* infiltration of *N. benthamiana* leaves. Scale bars: 50 µm. (**B**) Results of BiFC with agro-infiltrated *N. benthamiana* leaves showing interaction between NBR1-∆UBA and HSP90.1 in the epidermal layer. Scale bars: 25 µm. (**C**) Colocalization of NBR1-GFP and NBR1-∆UBA-GFP with ROF1-RFP. Imaging was done 2 d after *A. tumefaciens* infiltration of *N. benthamiana* leaves. Scale bars: 50 µm. (**D**) Results of BiFC with agro-infiltrated *N. benthamiana* leaves showing interaction between NBR1-∆UBA and ROF1 in the epidermal layer. Scale bars: 25 µm. (**E**) Fractionated (total, soluble and insoluble) extracts from Col-0 wild type (WT), *nbr1-2*, ROF1-RFP, *ROF1-RFP*/*nbr1-2* were loaded onto SDS-PAGE gels and subjected to silver staining for near-equal loading. (**F**) Results of immunodetection of HSP90 (left panel) and ROF1-RFP (right panel) in total (T), soluble (S) and insoluble (I) protein fractions of seedlings harvested after priming HS, 2 d into the recovery phase. Protein extracts were separated by centrifugation into soluble and insoluble/aggregated fractions and then subjected to immunoblot analysis using anti-RFP and anti-HSP90 antibodies, respectively. Note the higher protein levels of ROF1 and HSP90 protein in the soluble compared to insoluble fractions in *ROF1*/*nbr1-2* and *nbr1-2* seedlings compared to their corresponding controls.


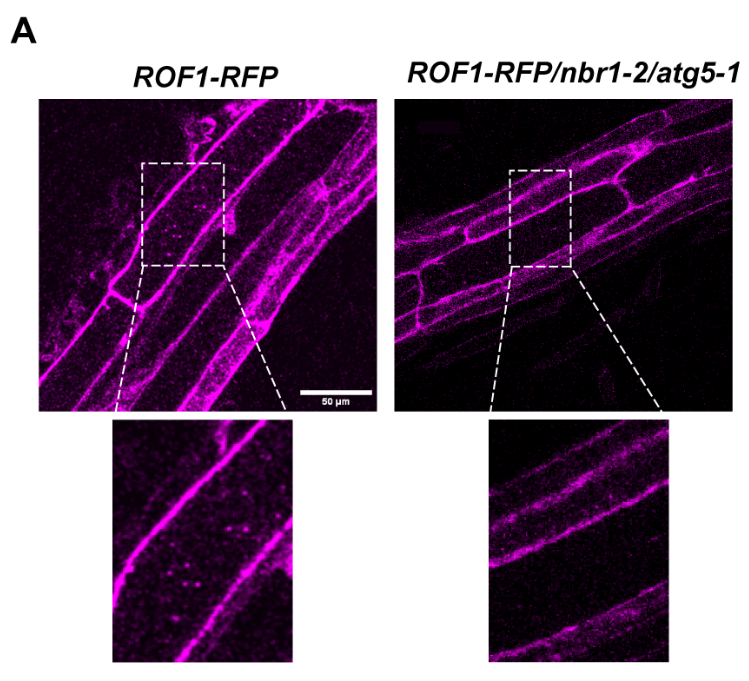


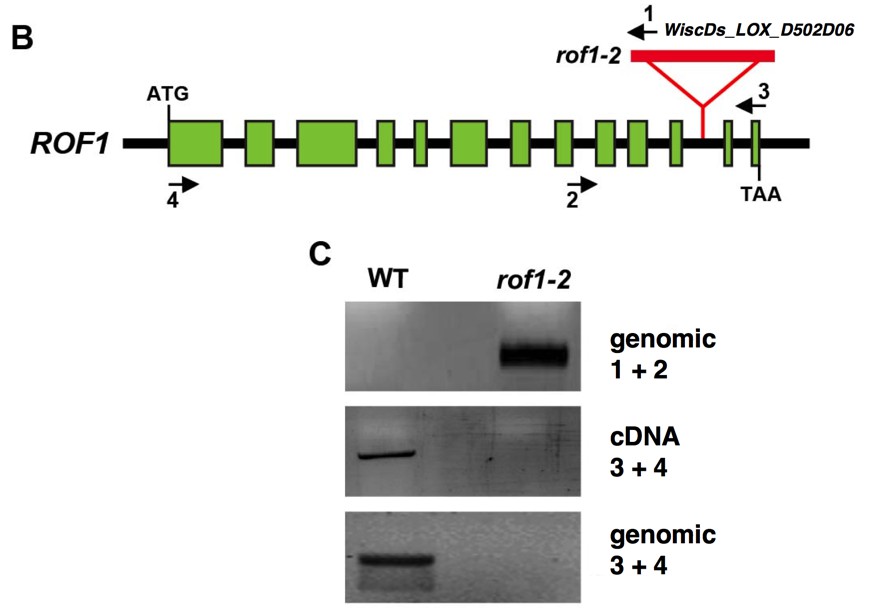


**Figure S5.** Comparison of ROF1 delivery to the vacuole between Col-0 and *atg5-1*/*nbr1-2* plants and identifying *rof1-2* knockout plants. (**A**) Roots (differentiation zone) of *pUBQ10:ROF1-RFP*/*Col-0* and *pUBQ10:ROF1-RFP*/*atg5-1*/*nbr1-2* seedlings were assessed by fluorescence confocal microscopy during the HS recovery phase (2 d after priming HS) following ConA (or DMSO control) treatment. Insets represent the delivery of ROF1 in the vacuole. Scale bars: 50 µm. (**B**) Schematic presentation of the *ROF1* gene in *Arabidopsis*, showing the T-DNA insertion position in *rof1-2*. (**C**) Upper panel, homozygous *rof1-2* plants were identified by PCR using a T-DNA-specific primer and a *ROF1* gene-specific primer (1+2). Middle panel, transcript analysis (end-point PCR) of *ROF1* in Col-0 and *rof1-2* plants using primers 3 and 4. Complementary DNA (cDNA) was used as a template for PCR. Note, *ROF1* transcript (1.6 kb) is absent in *rof1-2* plants, confirming that *rof1-2* is a null mutant. Lower panel, PCR-mediated amplification of *ROF1* full-length gene fragment using primers spanning the start and stop codons of the *ROF1* gene (3+4). Genomic DNA (gDNA) was used as a template for PCR. Notably, The *ROF1* gene fragment (2.8 kb) is absent in *rof1-2* gDNA.


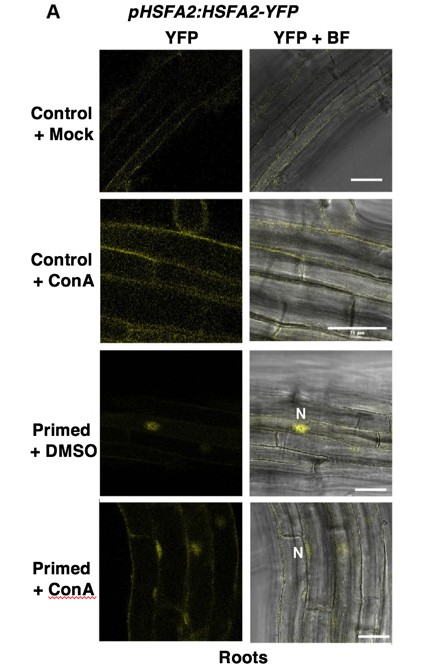


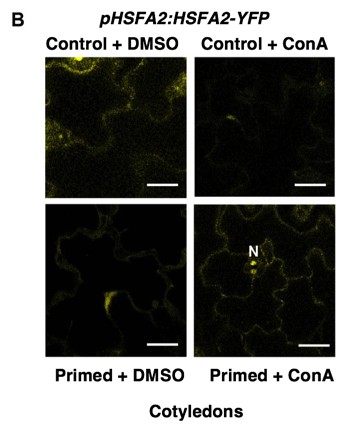


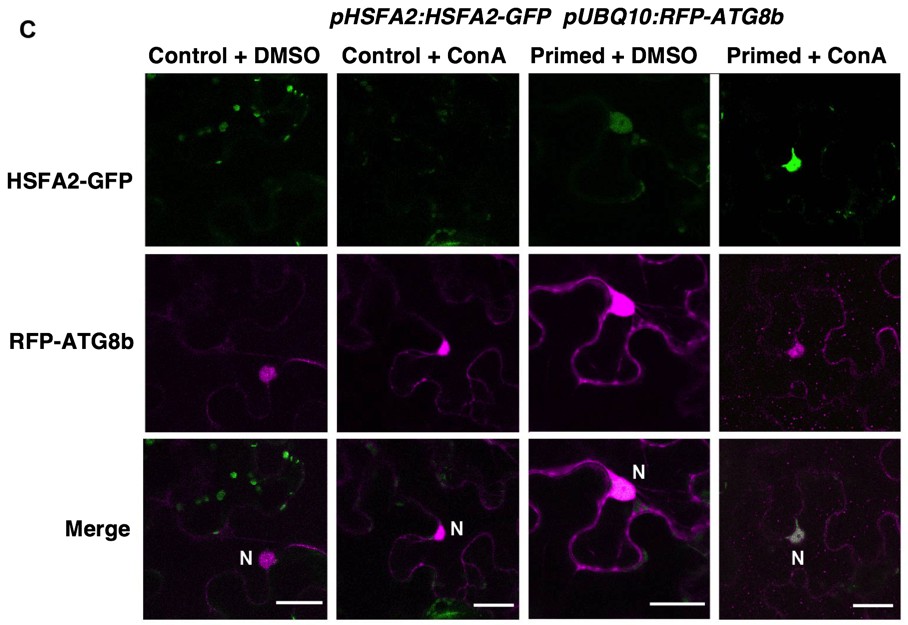


**Figure S6.** HSFA2 is not targeted by autophagy during the HS recovery phase. (**A**) Roots (differentiation zone) of *pHSFA2:HSFA2-YFP* seedlings were assessed by fluorescence confocal microscopy under control condition (unprimed) and after priming (2 d into the recovery phase), following ConA (or DMSO control) treatment. (**B**) Cotyledons of *pHSFA2:HSFA2-YFP* seedlings were assessed by fluorescence confocal microscopy under control condition (unprimed) and after priming (2 d into the recovery phase) following ConA (or DMSO control) treatment. (**C**) HSFA2 does not colocalize with RFP-ATG8b in the vacuoles. The *pUBQ10:RFP-ATG8b* and *pHSFA2:HSFA2-GFP* constructs were infiltrated into *Nicotiana benthamiana* leaves and subjected to priming HS treatment. Two days later, leaf sections were treated with DMSO control or ConA, and assessed by fluorescence confocal microscopy. Scale bar: 25 μm. N denotes nucleus.

Figure 2F


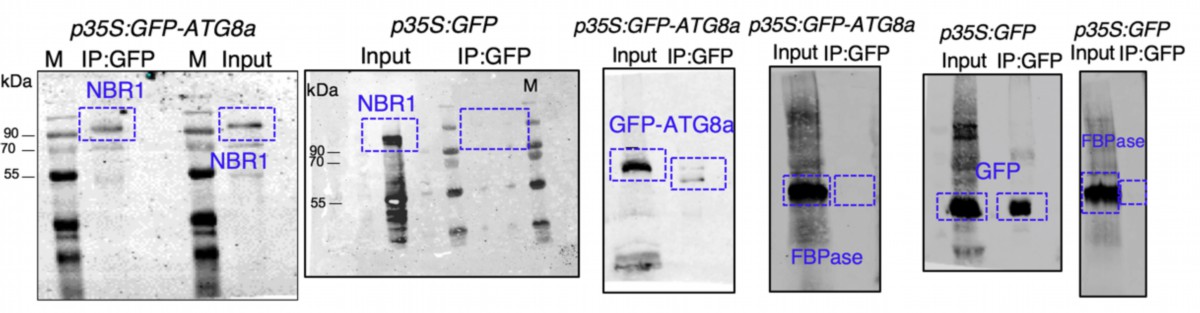


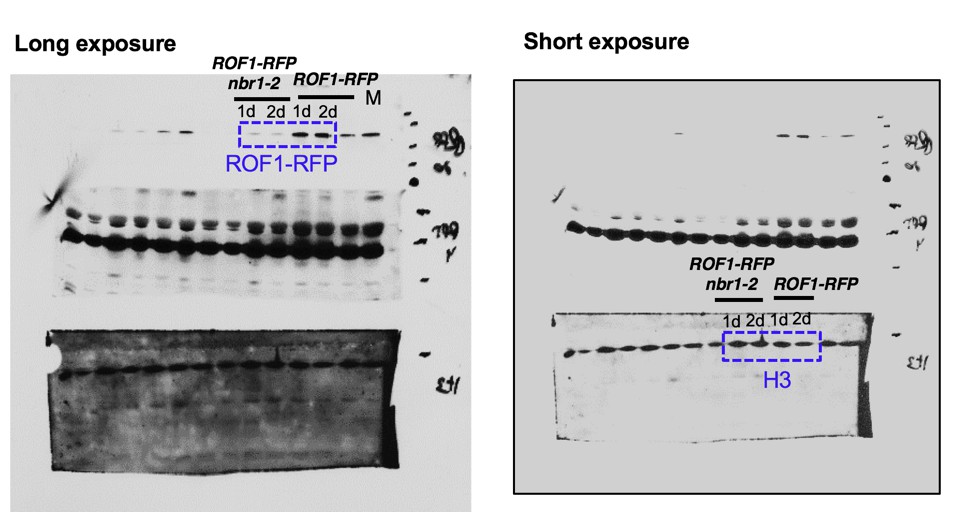


Figure 4I

Figure 3D

Figure 3E


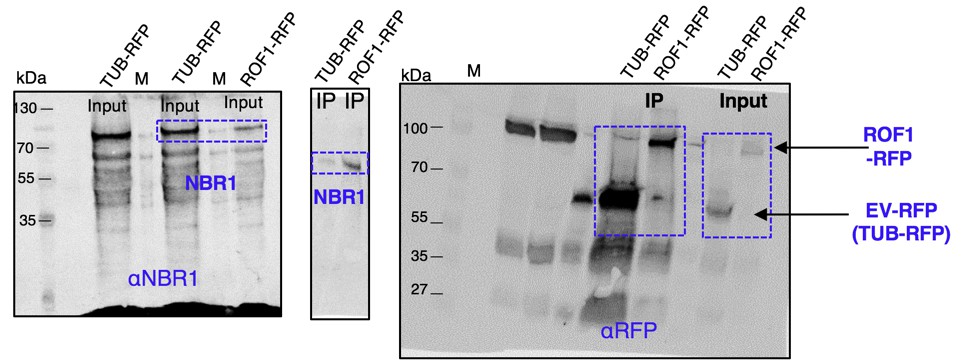


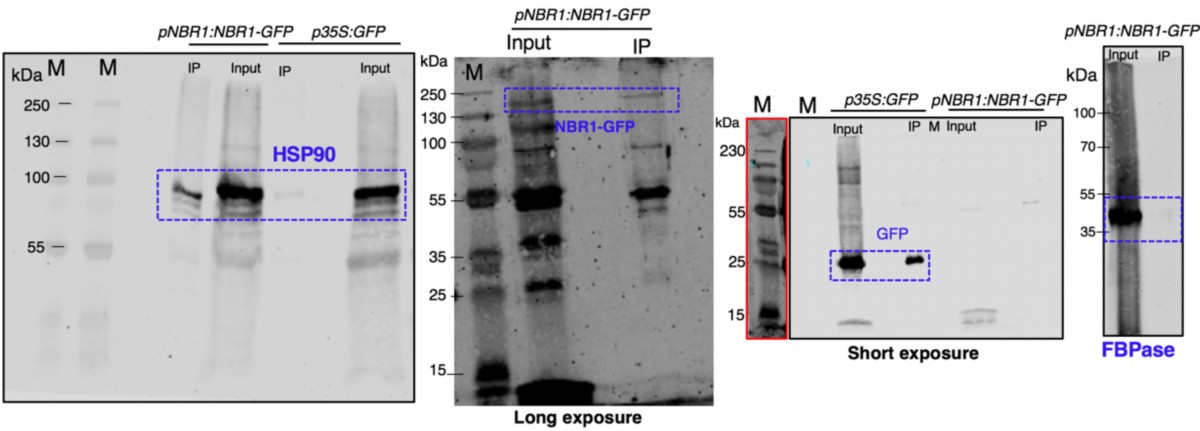


Figure 4J


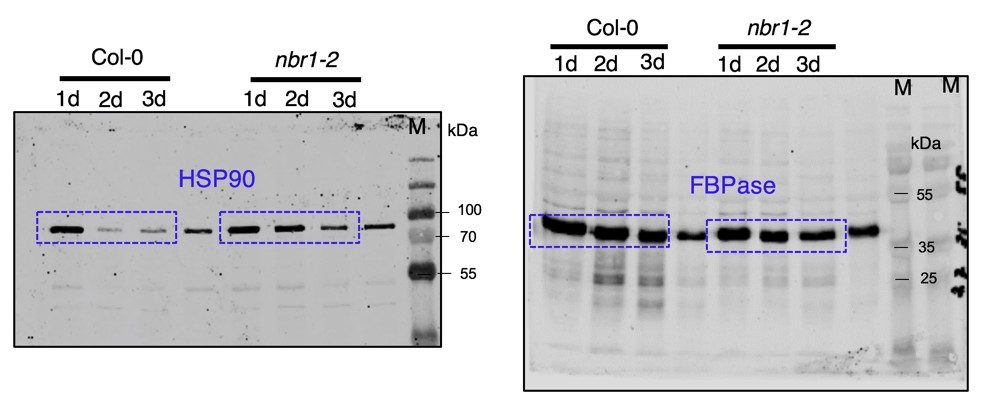


Figure 1D

Figure 2A


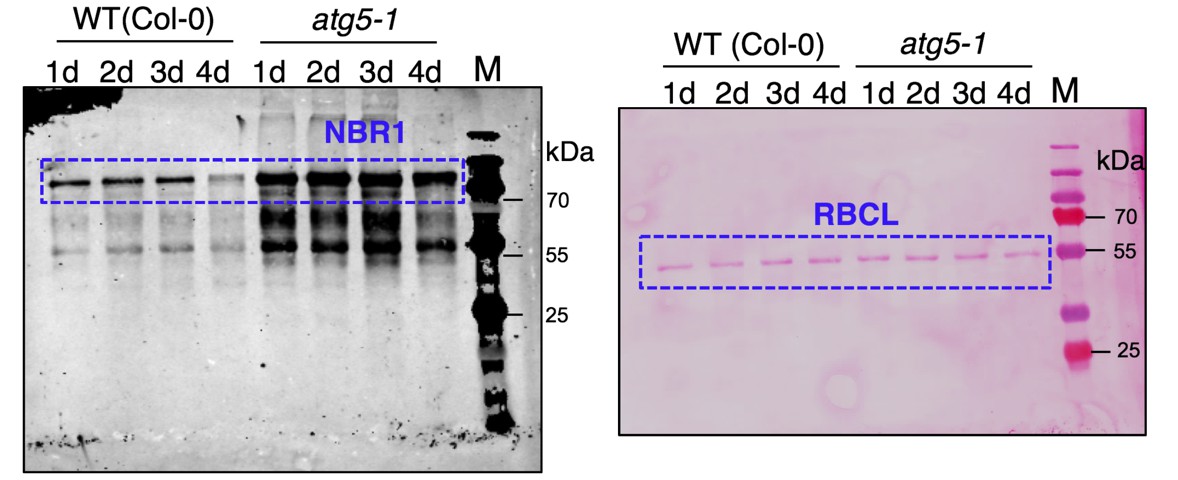


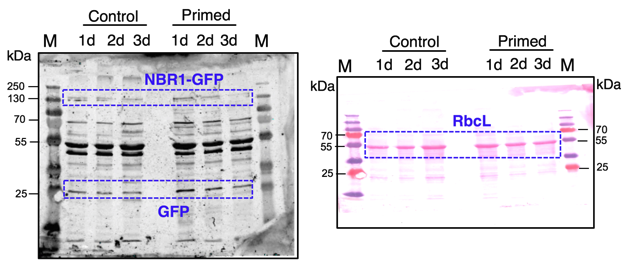


**RBCL**

Figure 5C

Figure 5B


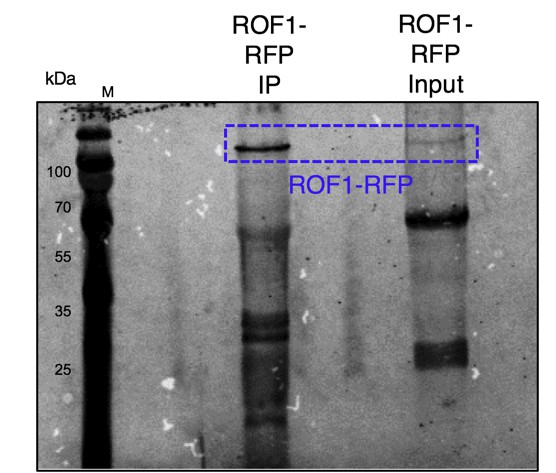


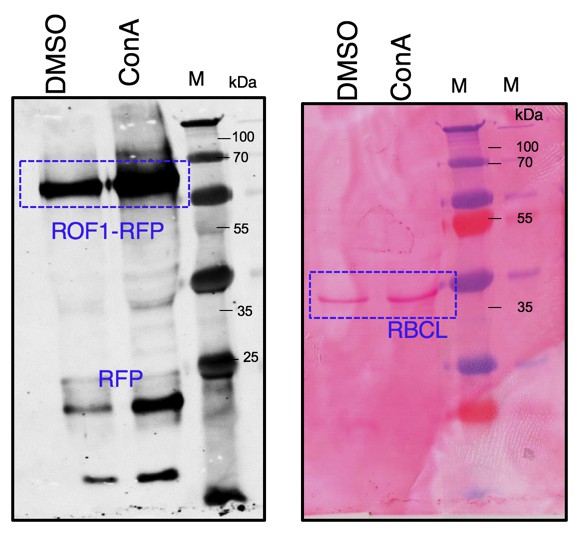


**Figure S7.** Uncropped images of the immunoblots used in the study. Labelling above each image indicates the corresponding figure in the main manuscript. M, molecular weight marker. Numbers left and right of images indicate protein molecular mass in kilodalton (kDa).

**Supplementary Materials, Videos**

**Video S1**. Accumulation of NBR1-GFP-labeled puncta in *Arabidopsis* cotyledons, at 2 d after priming upon ConA treatment.

**Video S2.** Colocalization of NBR1-GFP and RFP-ATG8b in *Arabidopsis* cotyledons, at 2 d after priming upon ConA treatment.

**Video S3.** Colocalization of NBR1-GFP and ROF1-RFP in *Arabidopsis* cotyledons, at 2 d after priming upon ConA treatment.

**Video S4.** Colocalization of NBR1-GFP and HSP90.1-RFP in *Arabidopsis* cotyledons, at 2 d after priming upon ConA treatment.

**Video S5a.** Colocalization of GFP-ATG8a and ROF1-RFP in the epidermal sections of *Arabidopsis* cotyledons, at 2 d after priming upon ConA treatment.

**Video S5b**. Colocalization of GFP-ATG8a and ROF1-RFP in *Arabidopsis* root sections, at 2 d after priming upon ConA treatment

**Video S6**. Accumulation of ROF-RFP-labeled puncta in *Arabidopsis* roots at 2 d after priming upon ConA treatment.

**Video S7**. Accumulation of HSP90.1-RFP-labeled puncta upon ConA treatment 2 d after infiltration in *N. benthamiana*.

**Video S8.** HSFA2-YFP does not accumulate in *Arabidopsis* root vacuoles at 2 d after priming upon ConA treatment.
